# Supplementary material for: Isofrequency spin-wave imaging using color center magnetometry for magnon spintronics
Source: Nat Commun. 2025 Dec 12;17:379. doi: 10.1038/s41467-025-67056-1 (PMC12796423; doi:10.1038/s41467-025-67056-1)
Supplement: Supplementary file 1 — Supplementary Information [file 41467_2025_67056_MOESM1_ESM.pdf]

Supplementary Information for

Isofrequency spin-wave imaging using color center magnetometry for magnon spintronics

Samuel Mañas-Valero,[1*] Yasmin C. Doedes,[1] Artem Bondarenko,[1] Michael Borst,[1] Samer Kurdi,[2] Thomas Poirier,[3] James H. Edgar,[3] Vincent Jacques,[4] Yaroslav M. Blanter,[1] Toeno van der Sar[1*]

1 Department of Quantum Nanoscience, Kavli Institute of Nanoscience, Delft University of Technology, Delft 2628CJ, the Netherlands

2 Institute of Photonics and Quantum Sciences, SUPA, Heriot-Watt University, Edinburgh EH14 4AS, United Kingdom

3 Tim Taylor Department of Chemical Engineering, Kansas State University, Kansas 66506, USA

4 Laboratoire Charles Coulomb, Université de Montpellier and CNRS, 34095 Montpellier, France

e-mail: S.ManasValero@tudelft.nl, T.vanderSar@tudelft.nl

This file contains the following information:

1. Methods....2
  1.1. hBN thin-layer fabrication....2
  1.2. Device fabrication....3
  1.3. Magnetic field control....4
  1.4. Fast Fourier Transform analysis....5
  1.5. Curling fittings....6
  1.6. Micromagnetic simulations....7
2. Spin-wave imaging....11
  2.1. Extended film....11
    2.1.1. $f = 3.44$ GHz (V_B center in hBN)....11
    2.1.2. $f = 2.87$ GHz (NV center in diamond)....14
  2.2. Half-plane film....17
    2.2.1. Angular dependence of the applied magnetic field: counterclockwise rotation 17
    2.2.2. Angular dependence of the applied magnetic field: clockwise rotation....19
    2.2.3. Microwave power dependence....22
    2.2.4. Laser power dependence....23
    2.2.5. NV-sample distance dependence....24
    2.2.6. Field strength dependence for $k \parallel B$....25
    2.2.7. Field strength dependence for $k \perp B$....26
    2.2.8. Spin wavelength evolution in a curling configuration....27
    2.2.9. Comparative between the theoretical and experimental spin wavelengths....29
3. Electron Spin Resonance (ESR)....30
  3.1. ESR spatial dependence for field applied parallel and perpendicular to the microstrip 30
  3.2. ESR spatial dependence for field applied perpendicular to the microstrip....32
  3.3. ESR angular dependence....33
4. References....35

1. Methods

1.1. hBN thin-layer fabrication

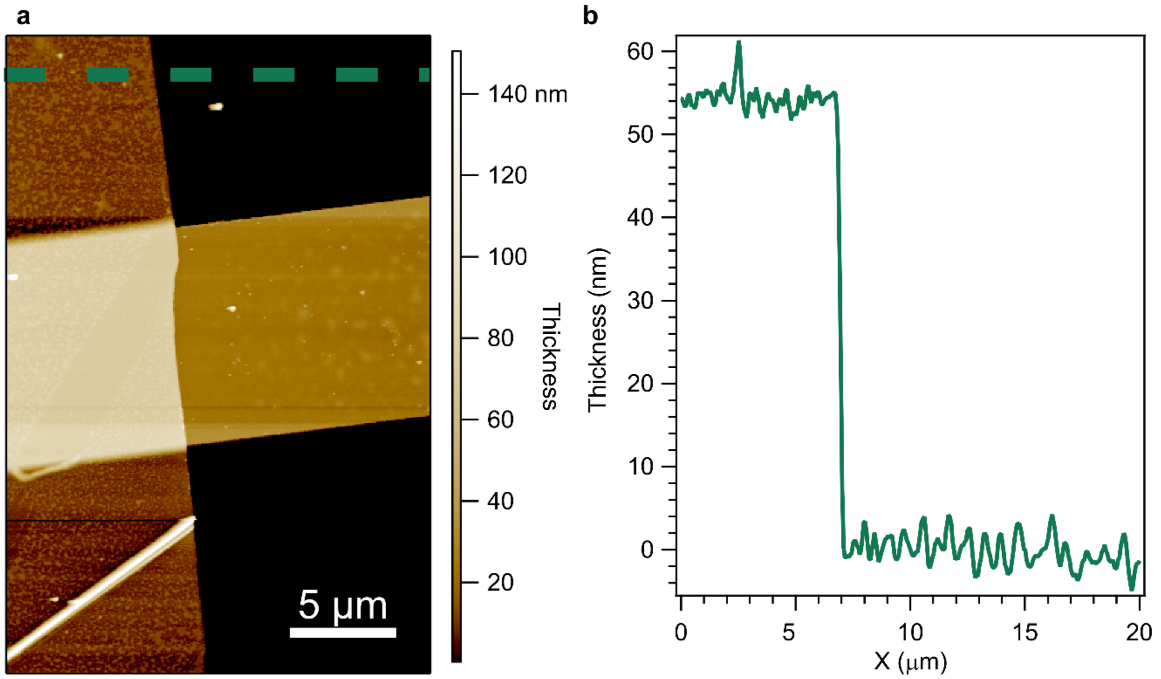

Supplementary Fig. 1. Atomic force microscopy image (a) of the hBN flake shown in the main text together with a height profile (b).

1.2. Device fabrication

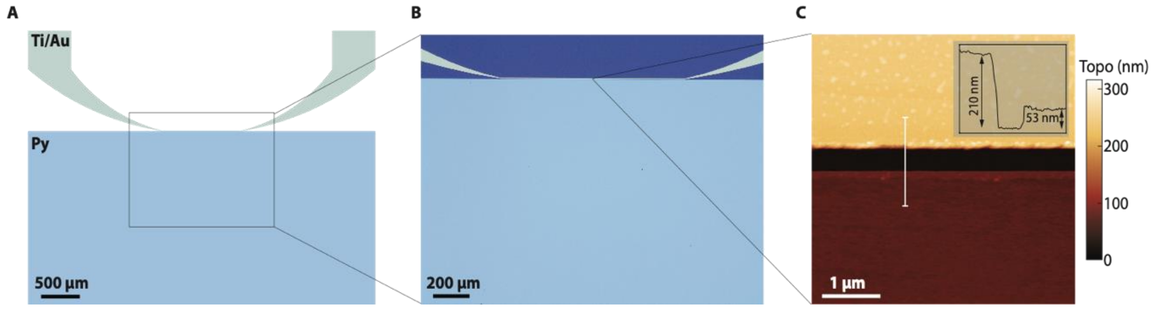

Supplementary Fig. 2. Overview of the sample: A permalloy magnetic half-plane next to a gold microstrip. A) Schematic, B) optical and C) atomic force image of our permalloy device which includes a titanium/gold (Ti/Au) microstrip and a rectangular permalloy (Py) structure. The inset in C is the height profile of the white line cut in the same figure.

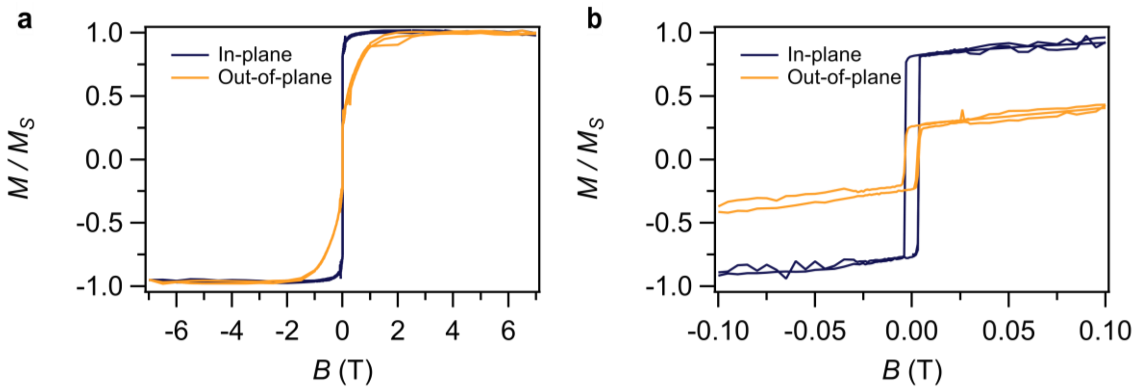

Supplementary Fig. 3.- Normalized magnetization of the permalloy films at room temperature for fields applied in-plane and out-of-plane.

1.3. Magnetic field control

We apply a magnetic field with a cylindrical magnet (Supermagnete S-05-14, dimensions length = 13.96 mm, diameter = 5 mm, and residual magnetization Br = 1.32-1.373 T). The magnet is mounted on a screw that is placed on a manual rotation stage (Thorlabs RP005). The magnet is oriented such that its axis is in the sample plane (XY-plane) for all possible angle rotations. The rotation angle can be tuned with an accuracy of 2 degrees. The rotation stage is placed on a tilt stage (Newport M-MM-2A), which in turn is placed on an XYZ-translation stage (Thorlabs MTS50-Z8). The magnet is aligned in the XY-plane by focusing the laser on the magnet and iteratively moving the magnet along X or Y, while adjusting the tilt based on the change in the laser spot size. Prior to a measurement, we magnetize the Py by decreasing the magnet-sample distance such that the magnetic field strength is above 33 mT.
The applied magnetic field is calibrated by measuring the ESR splitting of a NV diamond membrane (implantation dose 10^{13} at 54 keV). Those results are used for modelling the magnet using Magpylib (**Supplementary Fig. 4**).[1]

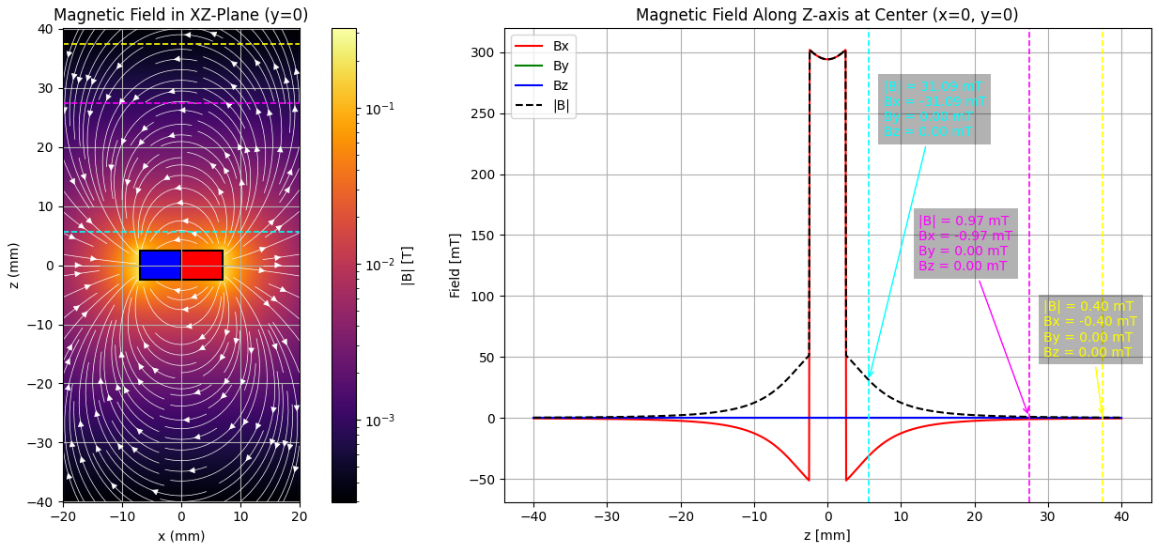

Supplementary Fig. 4. Calculation of the static magnetic field generated by the cylindrical magnet. Magnetic field simulation performed with Magpylib along the XZ plane (left panel) together with the corresponding field profile at x = 0 mm (right panel) with different representative z-values highlighted.

1.4. Fast Fourier Transform analysis

To extract the spin-wave wavelengths, we apply Fast Four Transforms (FFTs) to the spatial contrast maps using the software Gwyddion (https://gwyddion.net/). First, the Si and microstrip (X < 0 um) are cropped out of the image and the mean pixel value is subtracted from the cropped image. Then, 2D FFTs with a Hann window are applied. To find the wavelengths of the spin waves in the original images, the peaks along $k_y = 0$ are identified as the excited spin waves travel along X. The error in the extracted wavelength is set by the X range of the cropped image and the sampling frequency: $\Delta f = 2f_s/N$. We show a representative example for fields perpendicular ($\varphi = 0°$) and parallel ($\varphi = 90°$) to the microstrip in the **Supplementary Fig. 5**.

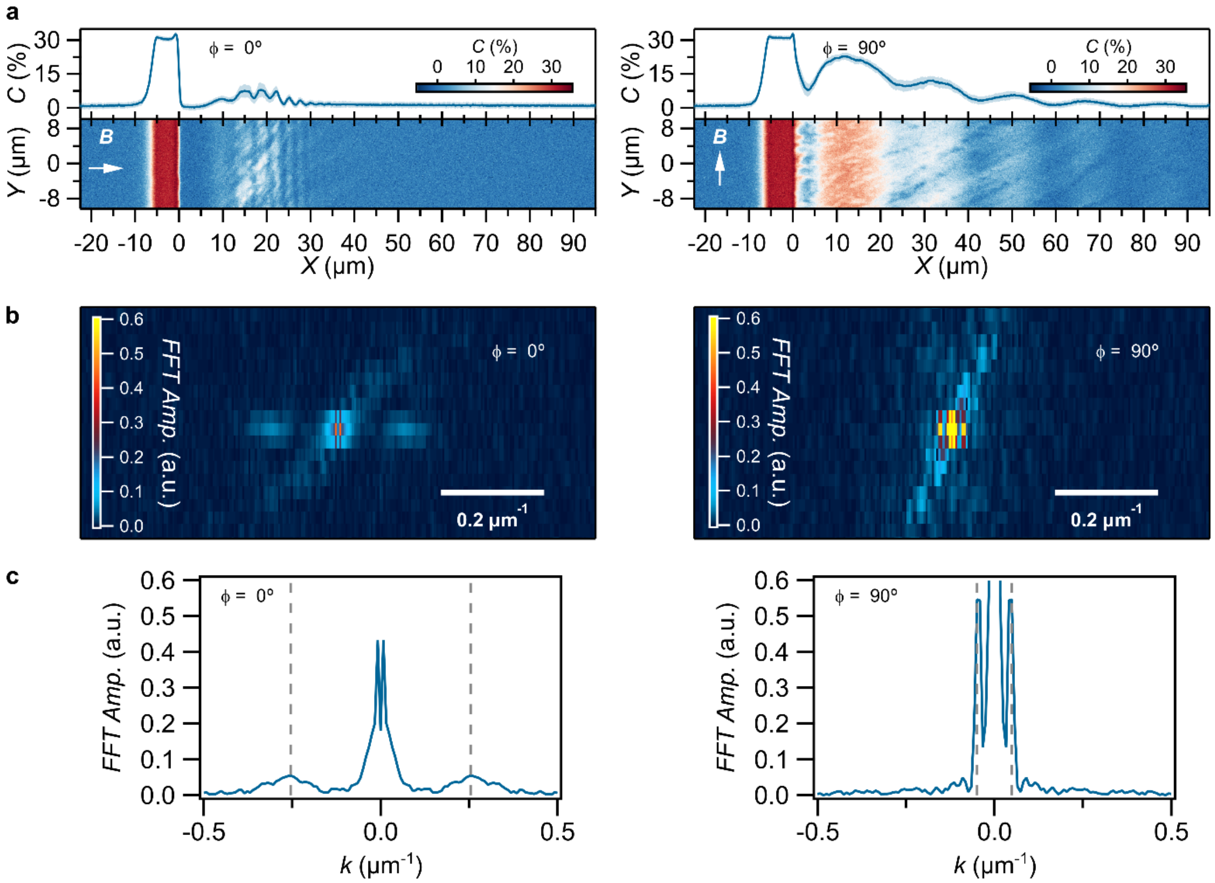

Supplementary Fig. 5. Examples for extracting the spin wavelengths from the spatial spin wave maps. FFT analysis for an applied magnetic field of 0.98 mT for $\varphi = 0°$ (left) and $\varphi = 90°$ (right). a) Spatial spin-wave maps and their average along the vertical direction. b) 2D FFT of the magnetic region (X > 0). c) FFT for $k_y = 0$ µm-1. The corresponding wavelength is indicated by a grey dashed line.

1.5. Curling fittings

The curling length of the spin reorientation when φ = 0° and 180° at B = 0.98 mT (**Fig. 3-5**) is found by fitting B_z vs. x based on a model of exponential decay of the magnetization projection along y, M_y, as a function of x by Hirono *et al.*:[2]

$$M_y = M_s t(\cos(\varphi_0) - \cos(\varphi_M)) * e^{-\frac{x}{x_0}} + \cos(\varphi_M)$$

$$M_x = \sqrt{1 - M_y^2}$$

where M_x is the magnetization projection along x, φ_0 = 0° is the magnetization angle with respect to y-axis at x = 0, φ_M is the (final) magnetization angle in the bulk, and x_0 is the curling length. φ_M and x_0 are the fitting parameters.

Following Dovzhenko *et al.*,[3] for an in-plane magnetized film that is spatially invariant along y, the resultant out-of-plane magnetic field is given by:

$$B_z = \frac{\mu_0 M_s t}{2\pi} \int f(x, d) \frac{\partial m_x}{\partial x} dx$$

where t is the thickness of the film and $f(x, d)$ is a filter function that accounts for the sensor-sample separation d = 500 nm.

The fit parameters are found to be x_0 = 19.1 ± 3.3 μm, φ_M = 69 ± 6° and x_0 = 11.2 ± 2.7 μm, φ_M = 46 ± 5°, for φ = 0° and 180°, respectively.

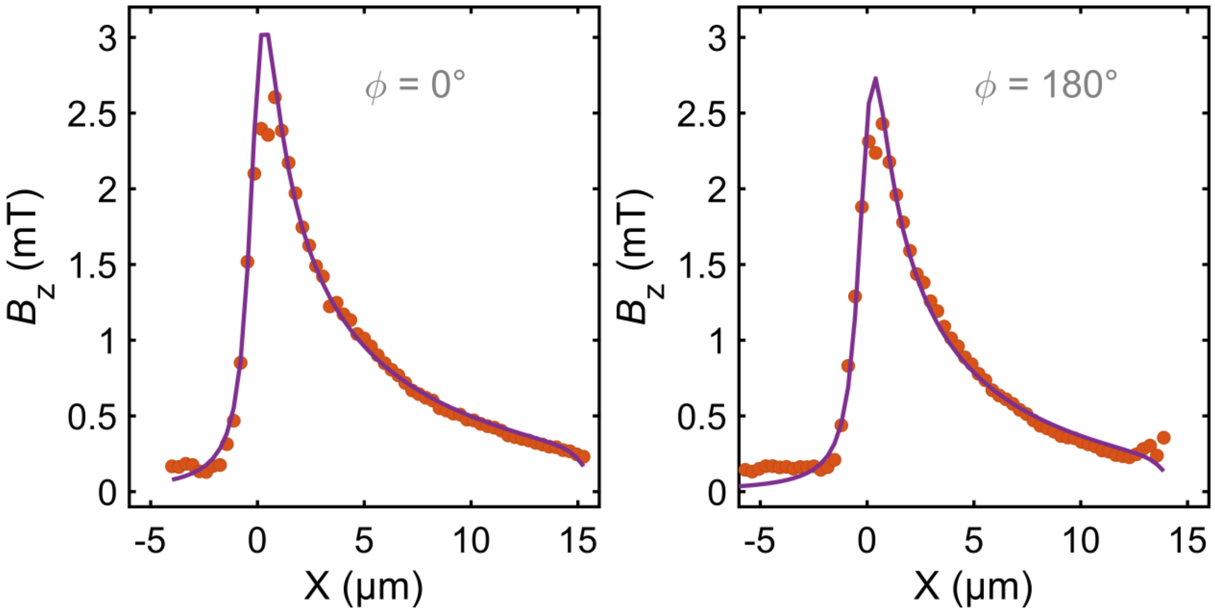

Supplementary Fig. 6. The out-of-plane magnetic field, B_z, as a function of x, with the data points (**Fig. 5**, averaged along y) in orange and fit based on the model described above in purple for φ = 0° and 180°. The fit is performed for all lines along y separately, the average of those results is shown.

1.6. Micromagnetic simulations

The magnetization of the film was simulated using the Mumax3 micromagnetics package. For the calculation we have converted the parameters obtained from the fit of the dispersion presented in **Fig. 4** of the main text and **Supplementary Section 1.5** into the following values for the software:

Supplementary Table 1.- Magnetization parameters employ for the micromagnetic simulations.

Parameter	Value
$A_{ex.}$	11.87 pJ/m
M_S	794 kA/m
K_U	792 J/m^3

The anisotropy constant is made to form an easy axis oriented in-plane of the film at the angle of 71.6 degrees with respect to the film edge normal.

An important part of recreating the magnetization textures is setting up a proper geometry where we can recreate the phenomenon at a much smaller scale than the one used in experiment because we need a very fine level of detail at the edge. Because of this we chose to simulate a strip which is finite in the X direction and has periodic boundary conditions in the Y direction.

The length in the X direction was carefully selected such that there is negligible interaction between the opposite edges (we simulated a 32 μm long segment). At the same time to have accurate demagnetizing field we selected the number of kernel repetitions along Y directions to be sufficiently large so that no artificial shape anisotropy is introduced (we did 50 PBC images at 8 μm width).

The seed magnetization before relaxing the system into a ground state is also of key importance for a clean capture of the metastable edge domain wall state. Because of a significant energy difference between the states, random seed magnetization on its own mostly anneals into a trivial curl state without a domain wall . One could seed the metastable state by rotating the external magnetic field, like in the experiment, however we found that numerically

we can achieve the same result either by rotating anisotropy axis, or by using a special seed state where the edge region (defined to be 500 nm deep) and the bulk have their initial magnetizations slightly offset from the X axis in different directions. In the **Supplementary Fig. 7** we show the dependence on the domain formation (domain and the relative field intensity between the doubly-peaked static-field profile) upon the anisotropy constants and saturation magnetization.

Mumax3 is not well-suited to calculate the stray fields from magnetization, since the distance between an NV center and the surface is much larger than the thickness of the film, and would impose a huge overhead. Instead, we do integration to obtain the stray field externally . Assuming that the magnetization is homogeneous both in depth and along the edge (Y axis) it is trivial to derive that the resulting out-of-plane stray field from the Green's function of the system (field of a magnetic dipole):[4]

$$B_z(x',z') = \frac{\mu_0 M_S}{4\pi} \int \frac{2t(x-x')(t+2z')m_x(x)dx}{((x-x')^2+z'^2)((x-x')^2+(z'+t)^2)}$$

where we denote the film thickness with t, and the film is centered around z=0. A detail view on the curling and domain formation is shown in **Fig. 5.c** and **Supplementary Fig. 8**.

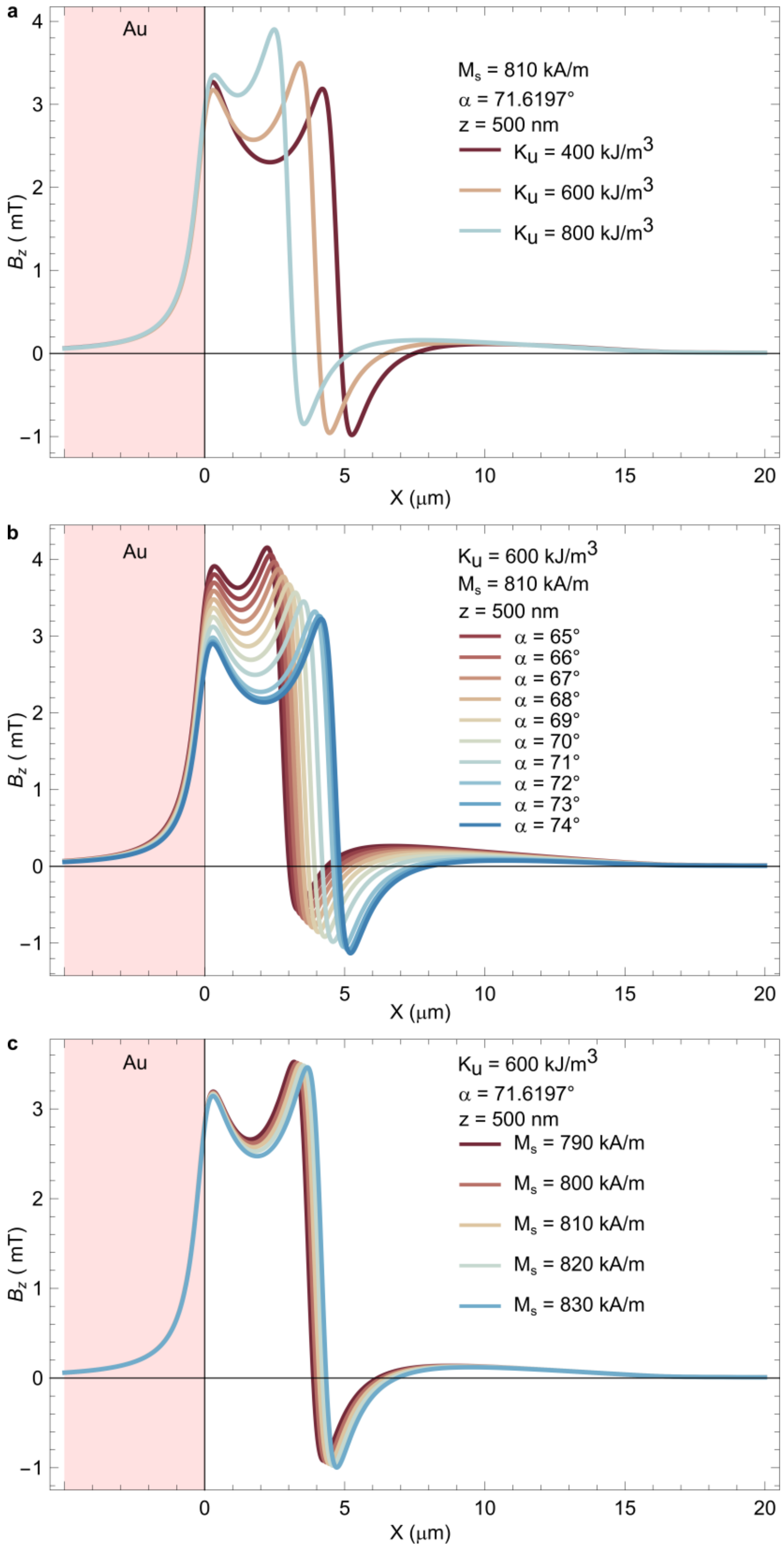

Supplementary Fig. 7. Dependence on the domain position and relative field intensity between the doubly-peaked static-field profile in the micromagnetic simulations. a) Uniaxial anisotropy constant (K_u) dependence. b) Anisotropy angle (α) dependence. c) Saturation magnetization (M_s) dependence. Red area indicate the location of the gold (Au) microstrip.

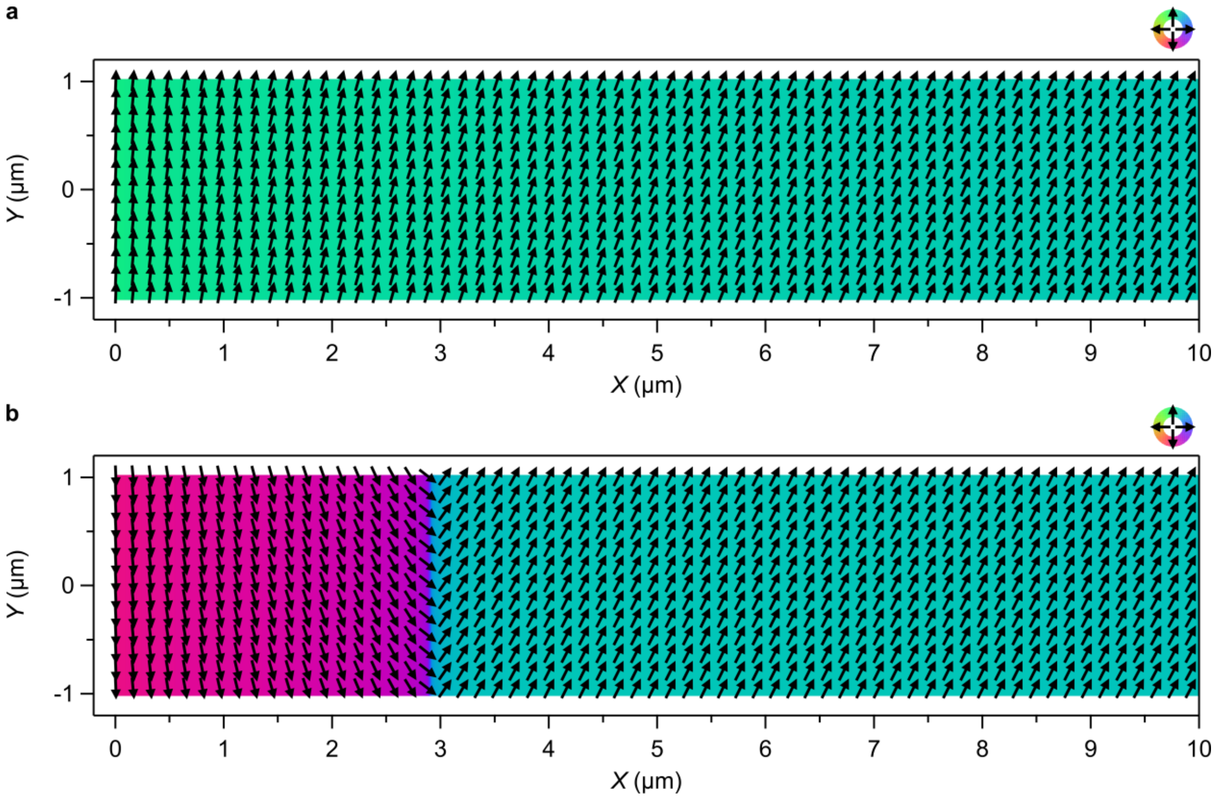

Supplementary Fig. 8. Micromagnetic calculations of the spatial spin textures for counterclockwise (a) and clockwise (b) rotation of the applied field, showing curling spin textures and the deterministic, field-rotation controlled nucleation of a domain wall. X = 0 corresponds to the film edge, as shown in **Fig. 5**.

2. Spin-wave imaging

In this section, we show the spatial contrast maps underlying the data shown in the main text.

2.1. Extended film

2.1.1. *f* = 3.44 GHz (V_B center in hBN)

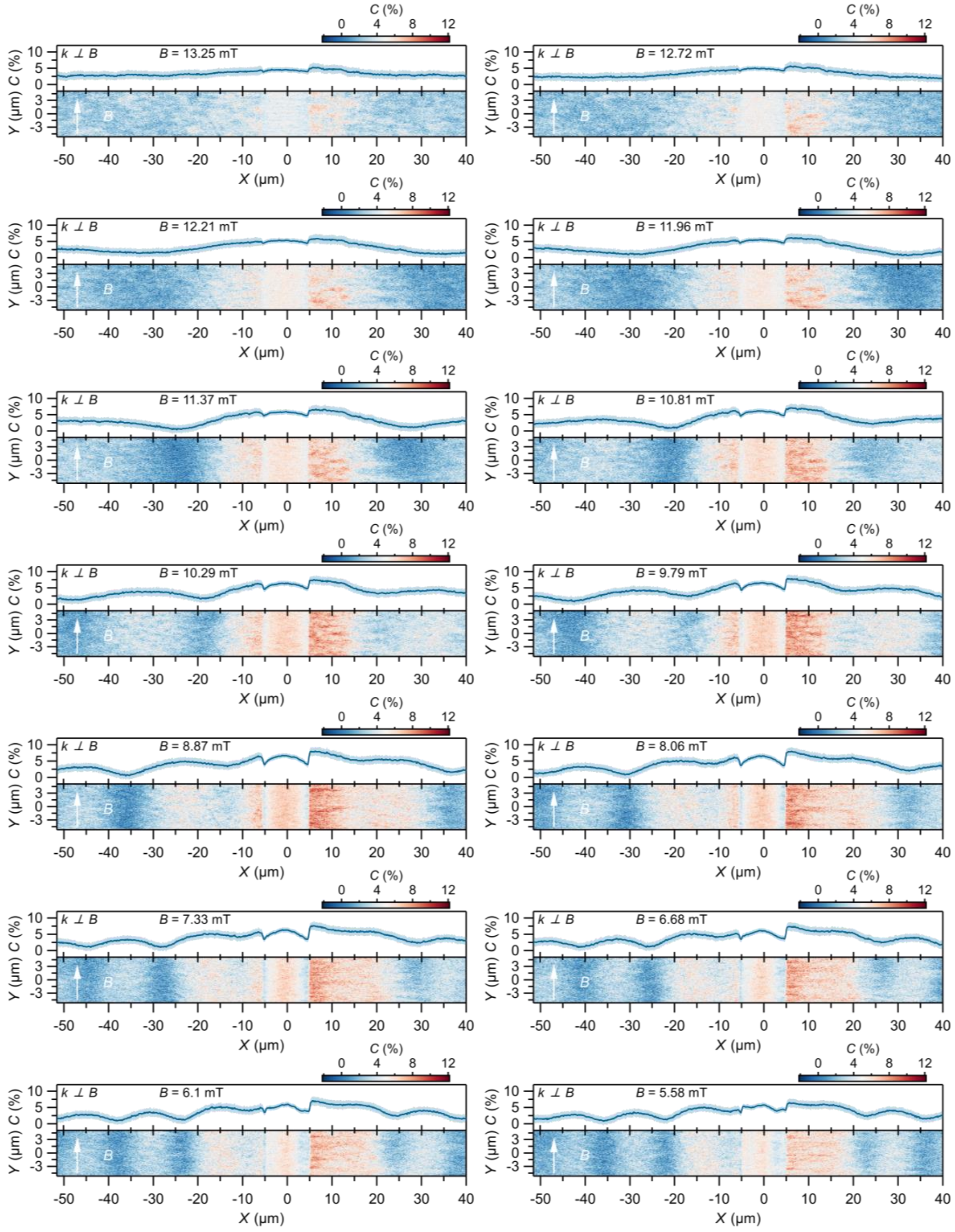

Supplementary Fig. 9. Spin-wave contrast spatial maps (bottom panel) and average profile along Y (top panel) for different external in-plane magnetic fields (denoted in every panel) applied magnetic field directions parallel to the microstrip (φ=270°). The microstrip correspond to ∈ [-5, 5] μm. The shaded area in the top panels corresponds to the standard deviation. The orientation of the applied magnetic field is denoted with a white arrow. The spin-waves are measured in a bimodal scheme with f = 3.44 GHz.

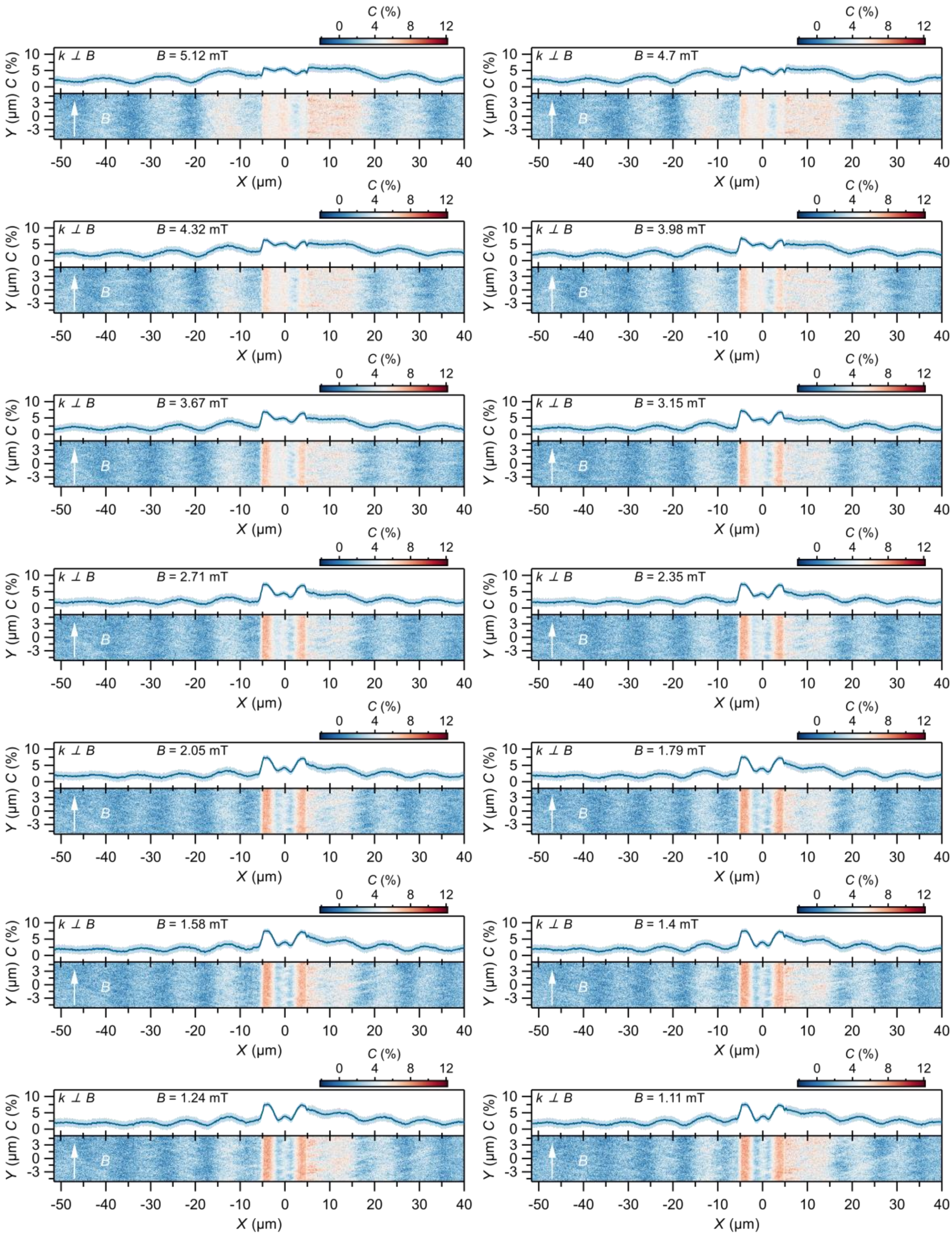

Supplementary Fig. 10. Spin-wave contrast spatial maps (bottom panel) and average profile along Y (top panel) for different external in-plane magnetic fields (denoted in every panel) applied magnetic field directions parallel to the microstrip (φ=270°). The microstrip correspond to ∈ [-5, 5] µm. The shaded area in the top panels corresponds to the standard deviation. The orientation of the applied magnetic field is denoted with a white arrow. The spin-waves are measured in a bimodal scheme with f = 3.44 GHz.

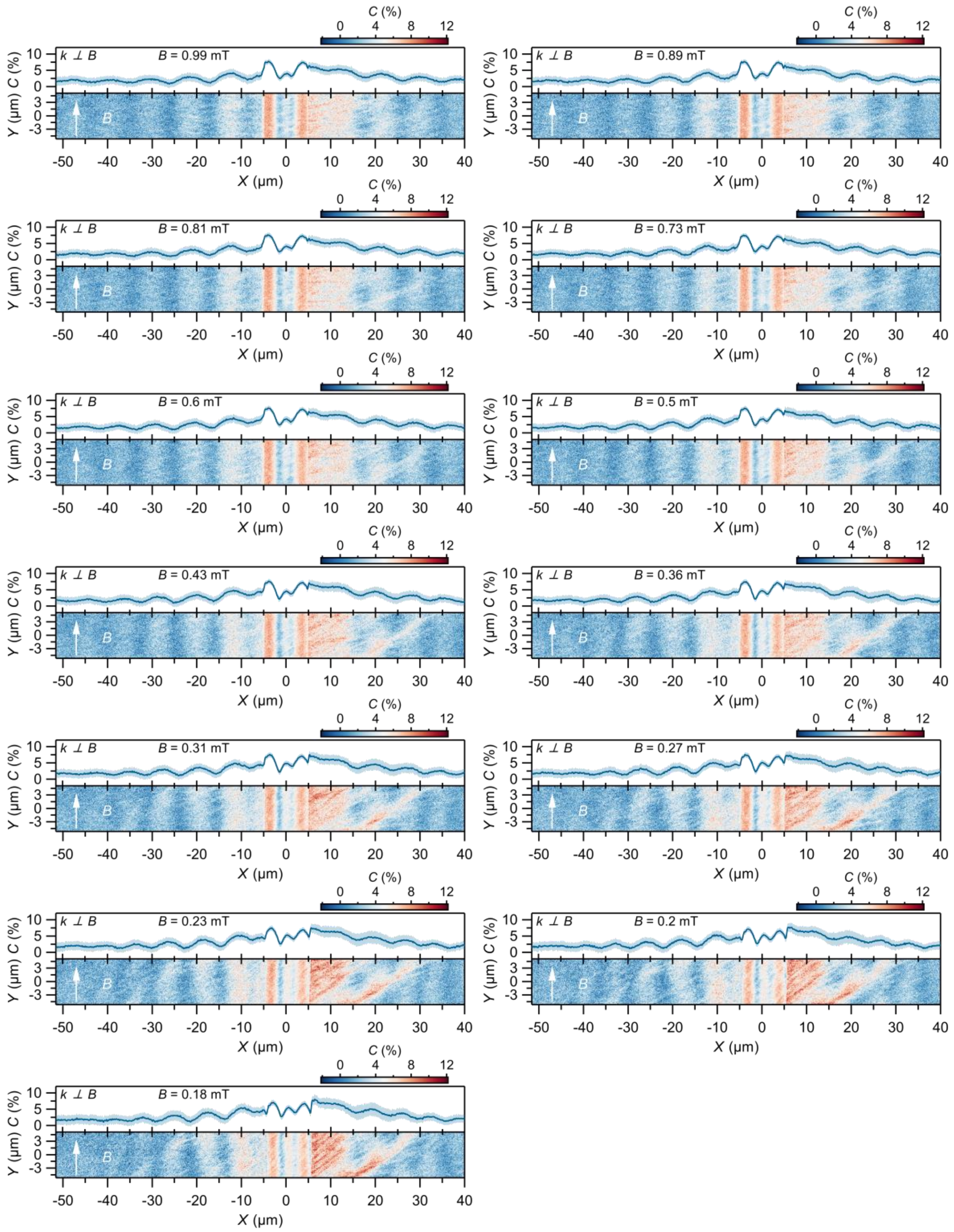

Supplementary Fig. 11. Spin-wave contrast spatial maps (bottom panel) and average profile along Y (top panel) for different external in-plane magnetic fields (denoted in every panel) applied magnetic field directions parallel to the microstrip (φ=270°). The microstrip correspond to ∈ [-5, 5] μm. The shaded area in the top panels corresponds to the standard deviation. The orientation of the applied magnetic field is denoted with a white arrow. The spin-waves are measured in a bimodal scheme with f = 3.44 GHz.

2.1.2. *f* = 2.87 GHz (NV center in diamond)

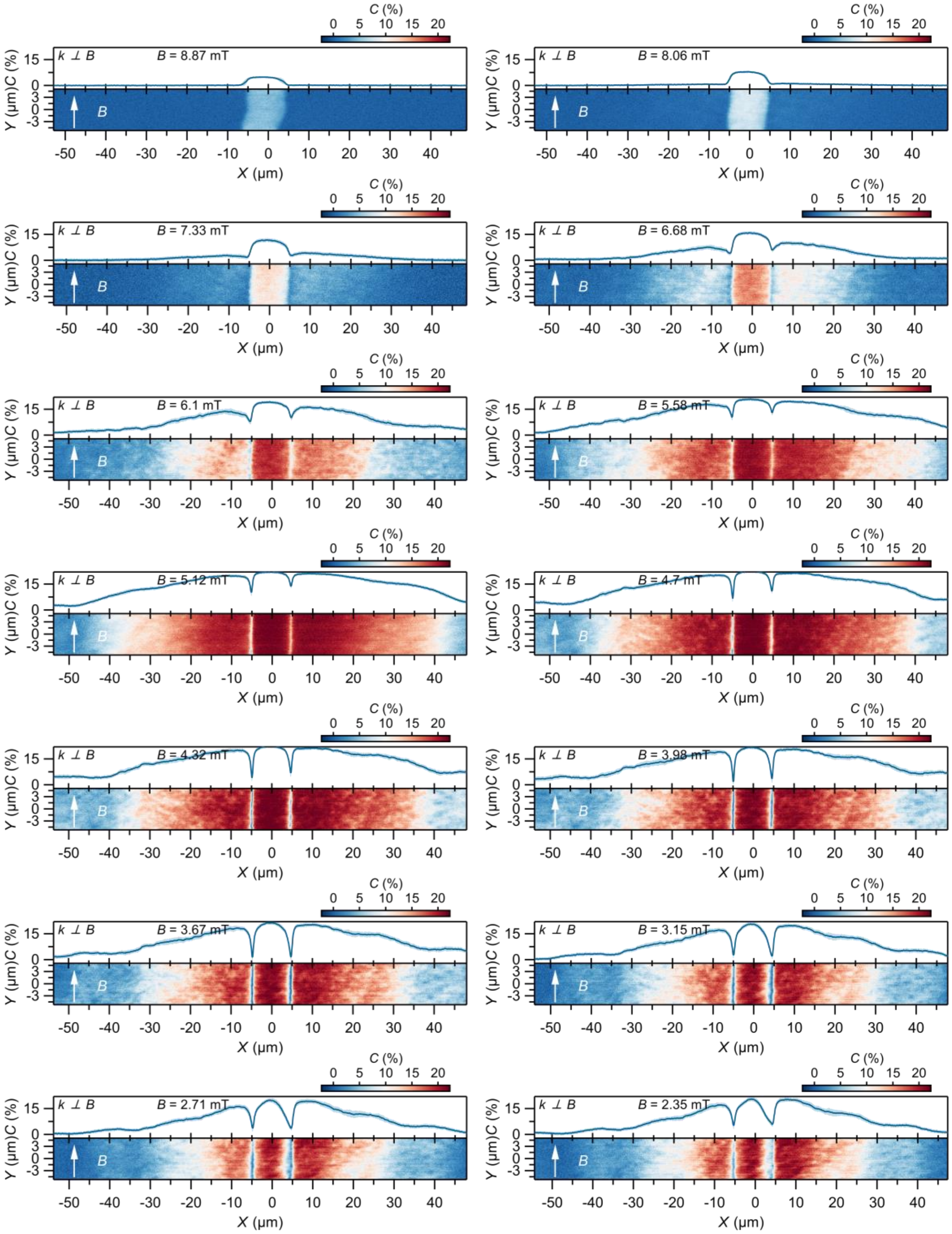

Supplementary Fig. 12. Spin-wave contrast spatial maps (bottom panel) and average profile along Y (top panel) for different external in-plane magnetic fields (denoted in every panel) applied magnetic field directions parallel to the microstrip (φ=270°). The microstrip correspond to ∈ [-5, 5] μm. The shaded area in the top panels corresponds to the standard deviation. The orientation of the applied magnetic field is denoted with a white arrow. The spin-waves are measured in a bimodal scheme with f = 2.87 GHz.

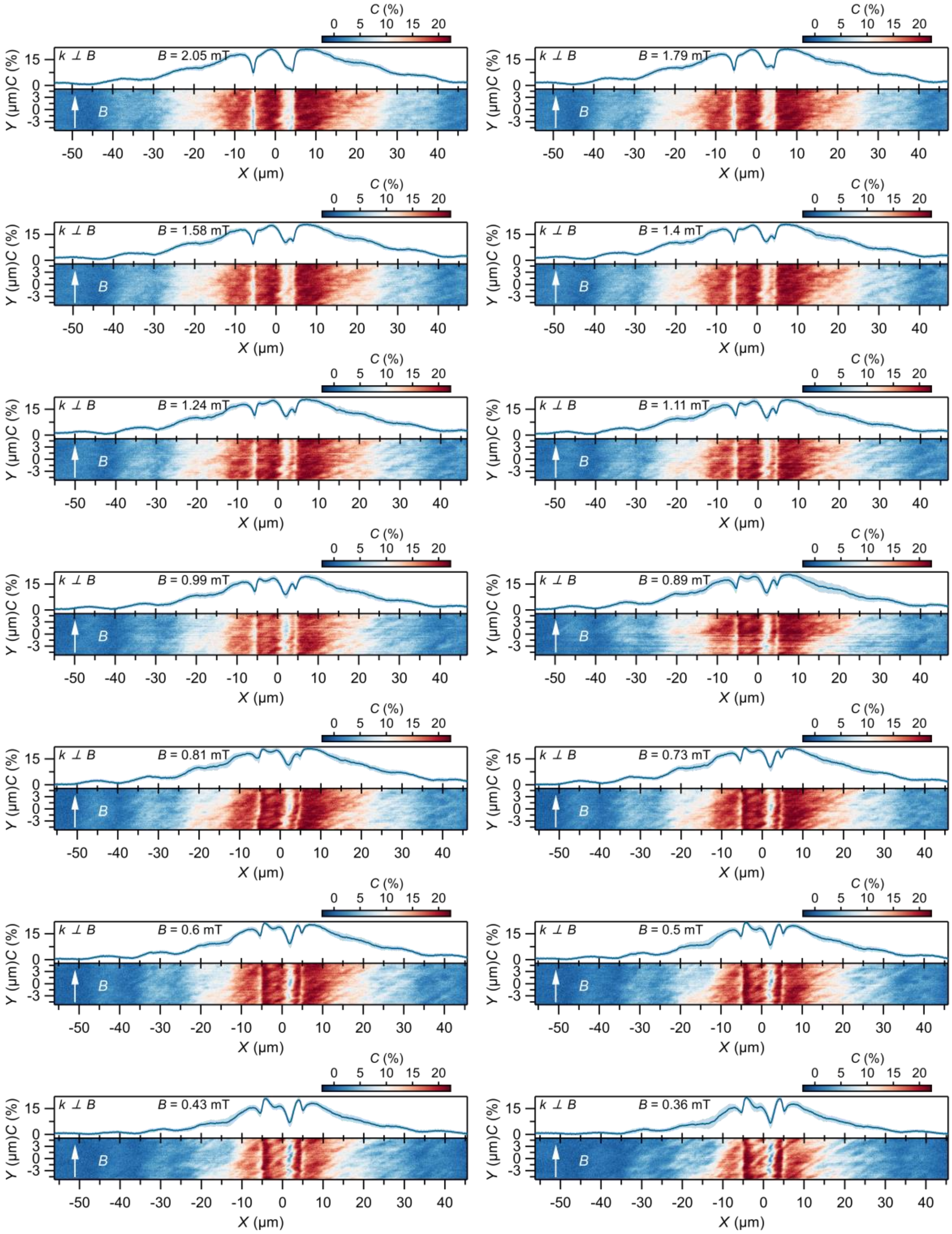

Supplementary Fig. 13. Spin-wave contrast spatial maps (bottom panel) and average profile along Y (top panel) for different external in-plane magnetic fields (denoted in every panel) applied magnetic field directions parallel to the microstrip (φ=270°). The microstrip correspond to ∈ [-5, 5] μm. The shaded area in the top panels corresponds to the standard deviation. The orientation of the applied magnetic field is denoted with a white arrow. The spin-waves are measured in a bimodal scheme with f = 2.87 GHz.

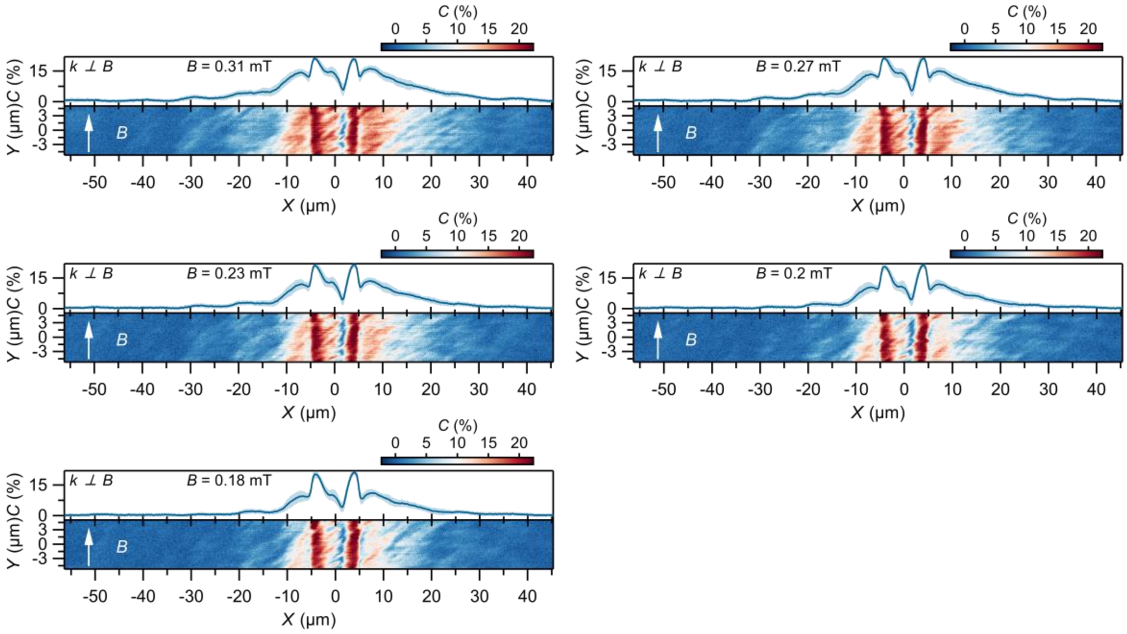

Supplementary Fig. 14. Spin-wave contrast spatial maps (bottom panel) and average profile along Y (top panel) for different external in-plane magnetic fields (denoted in every panel) applied magnetic field directions parallel to the microstrip (φ=270°). The microstrip correspond to ∈ [-5, 5] μm. The shaded area in the top panels corresponds to the standard deviation. The orientation of the applied magnetic field is denoted with a white arrow. The spin-waves are measured in a bimodal scheme with f = 2.87 GHz.

2.2. Half-plane film

2.2.1. Angular dependence of the applied magnetic field: counterclockwise rotation

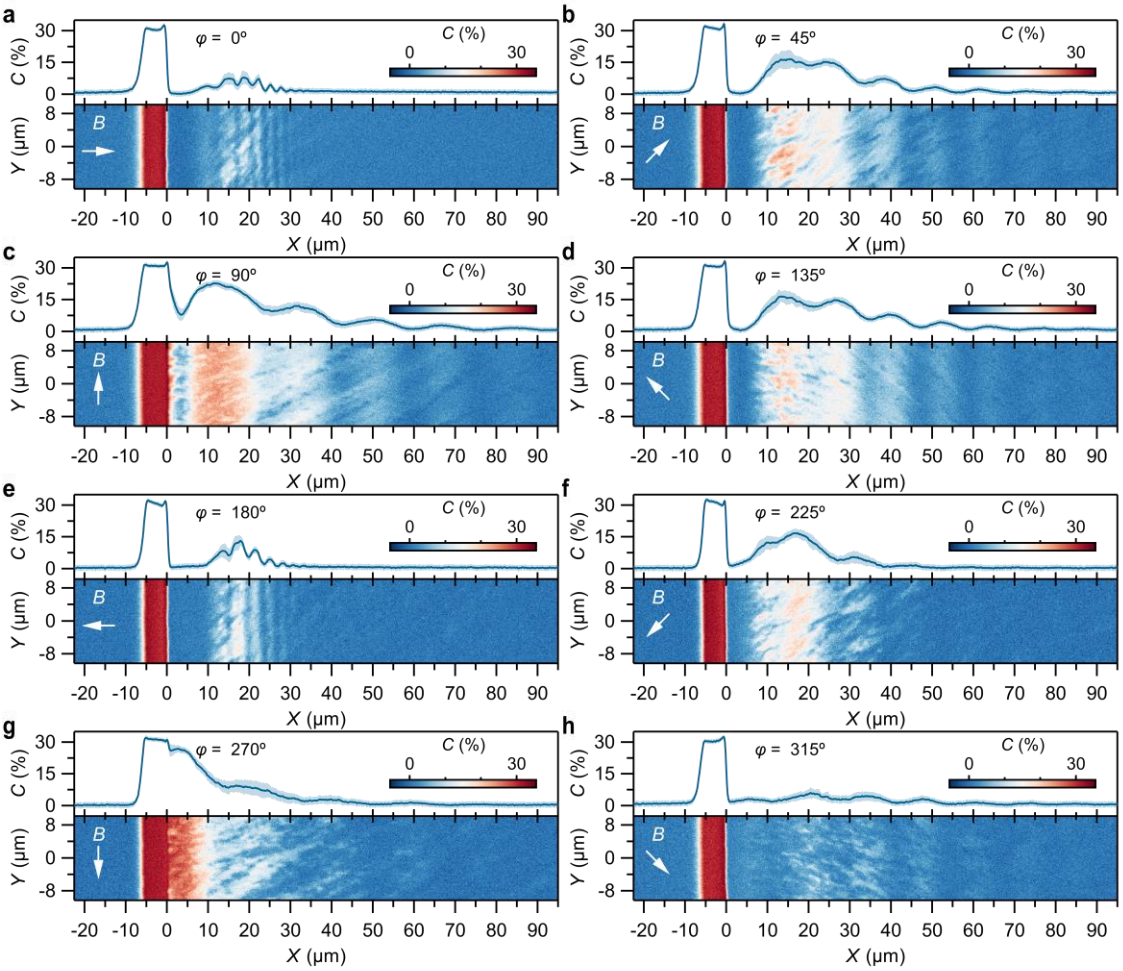

Supplementary Fig. 15. Spin-wave contrast spatial maps (bottom panel) and average profile along Y (top panel) for different in-plane applied magnetic field directions (φ). Silicon substrate, microstrip and Py film correspond to X < -5 µm, -5 < X < 0 µm, and X > 0 µm, respectively. The shaded area in the top panels corresponds to the standard deviation. The orientation of the applied magnetic field is denoted with a white arrow. The spin-waves are measured in a bimodal scheme with f = 2.869 GHz and an external applied magnetic field of 0.98 mT.

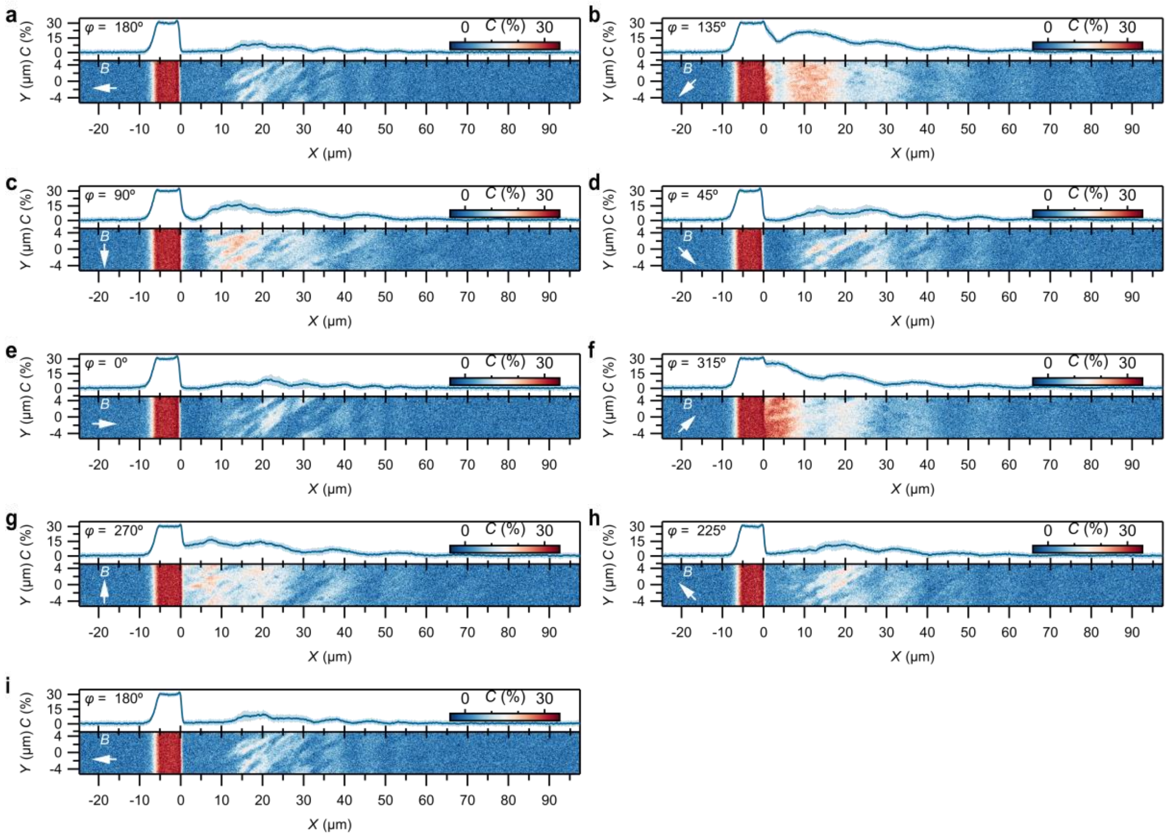

Supplementary Fig. 16. Spin-wave contrast spatial maps (bottom panel) and average profile along Y (top panel) for different in-plane applied magnetic field directions (φ). Silicon substrate, microstrip and Py film correspond to X < -5 µm, -5 < X < 0 µm, and X > 0 µm, respectively. The shaded area in the top panels corresponds to the standard deviation. The orientation of the applied magnetic field is denoted with a white arrow. The spin-waves are measured in a bimodal scheme with f = 2.869 GHz and an external applied magnetic field of 0.39 mT.

2.2.2. Angular dependence of the applied magnetic field: clockwise rotation

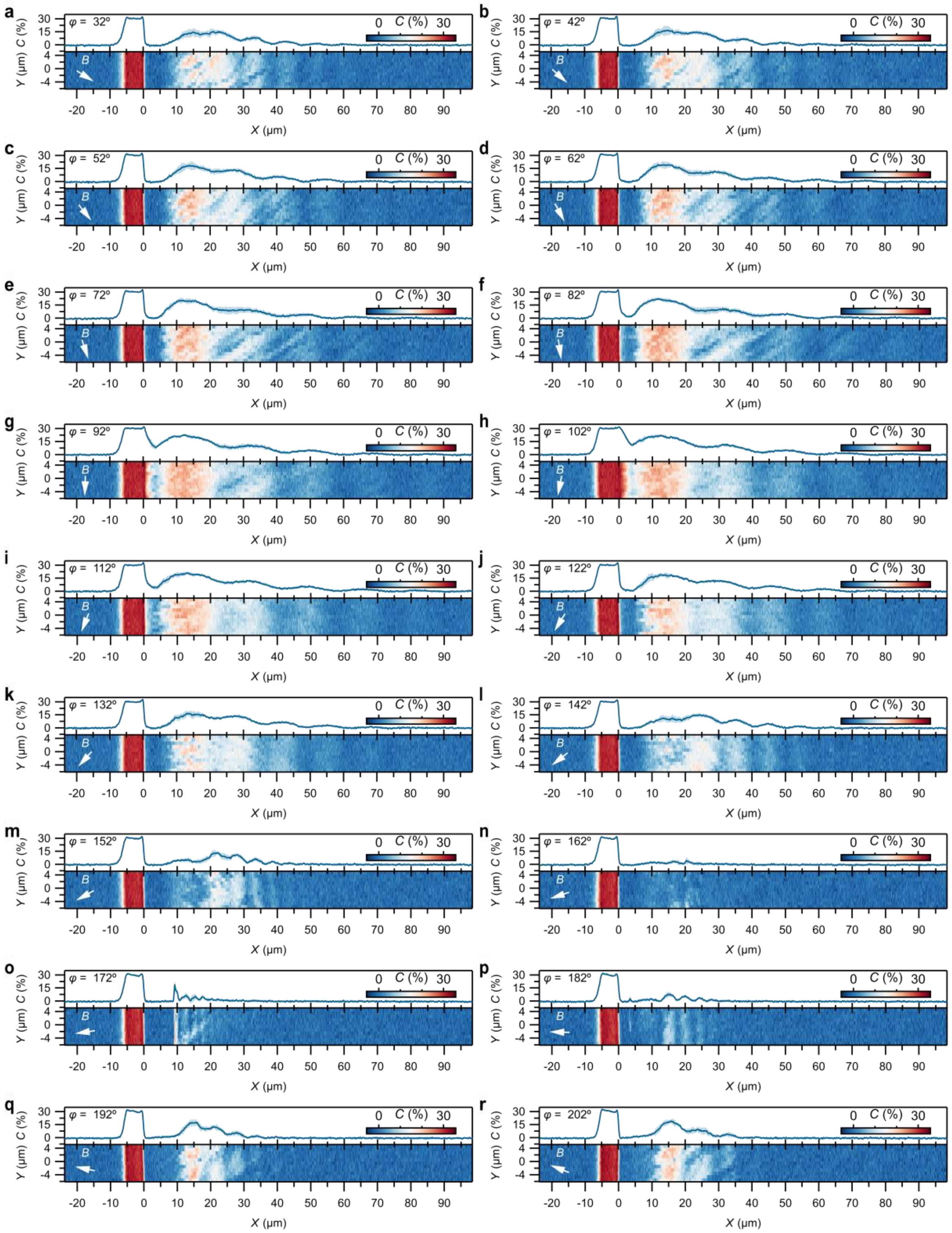

Supplementary Fig. 17. Spin-wave contrast spatial maps (bottom panel) and average profile along Y (top panel) for different in-plane applied magnetic field directions (φ). Silicon substrate, microstrip and Py film correspond to X < -5 μm, -5 < X < 0 μm, and X > 0 μm, respectively. The shaded area in the top panels corresponds to the standard deviation. The orientation of the applied magnetic field is denoted with a white arrow. The spin-waves are measured in a bimodal scheme with f = 2.869 GHz and an external applied magnetic field of 0.98 mT.

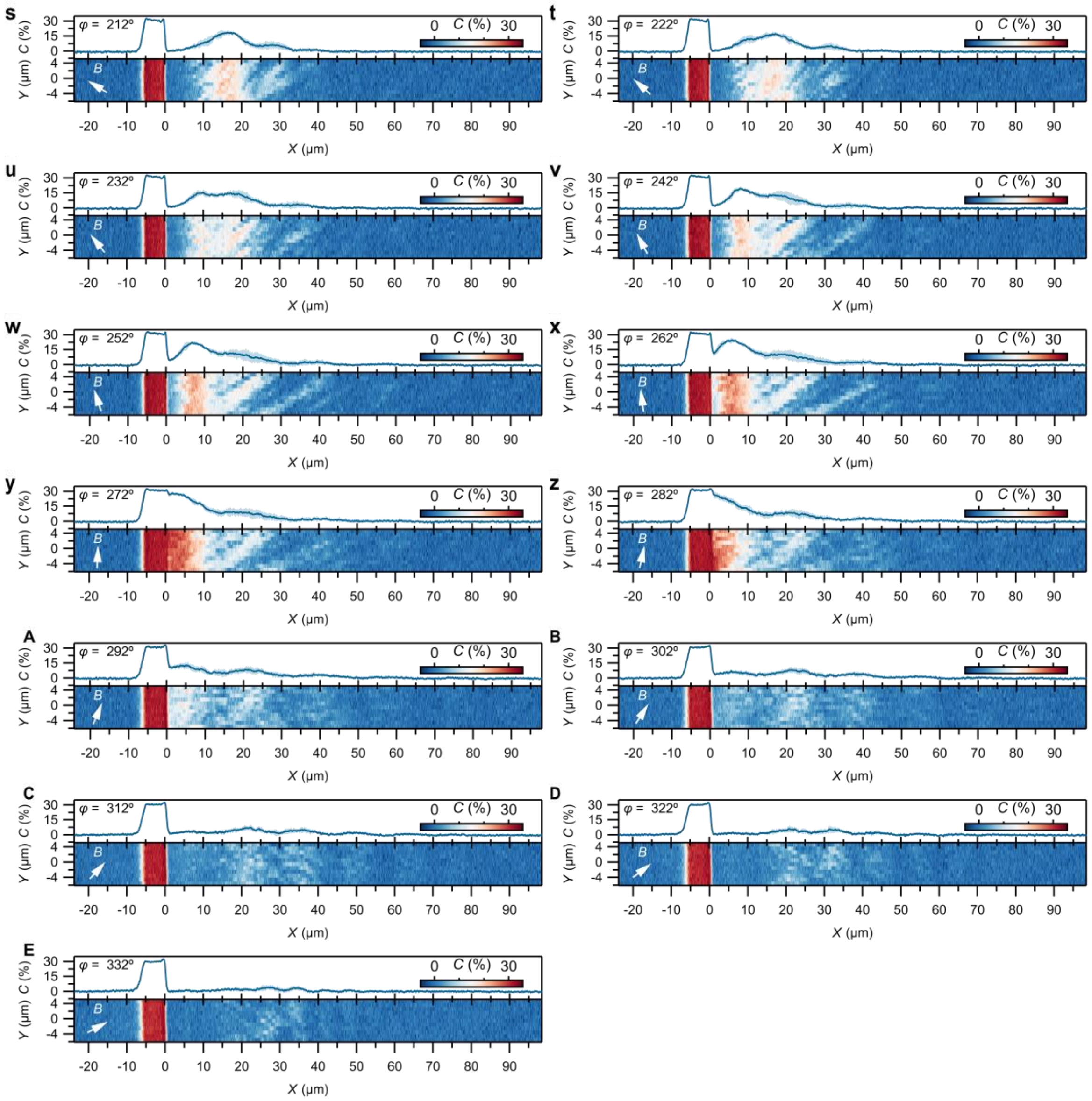

Supplementary Fig. 18. Spin-wave contrast spatial maps (bottom panel) and average profile along Y (top panel) for different in-plane applied magnetic field directions (φ). Silicon substrate, microstrip and Py film correspond to X < -5 μm, -5 < X < 0 μm, and X > 0 μm, respectively. The shaded area in the top panels corresponds to the standard deviation. The orientation of the applied magnetic field is denoted with a white arrow. The spin-waves are measured in a bimodal scheme with f = 2.869 GHz and an external applied magnetic field of 0.98 mT.

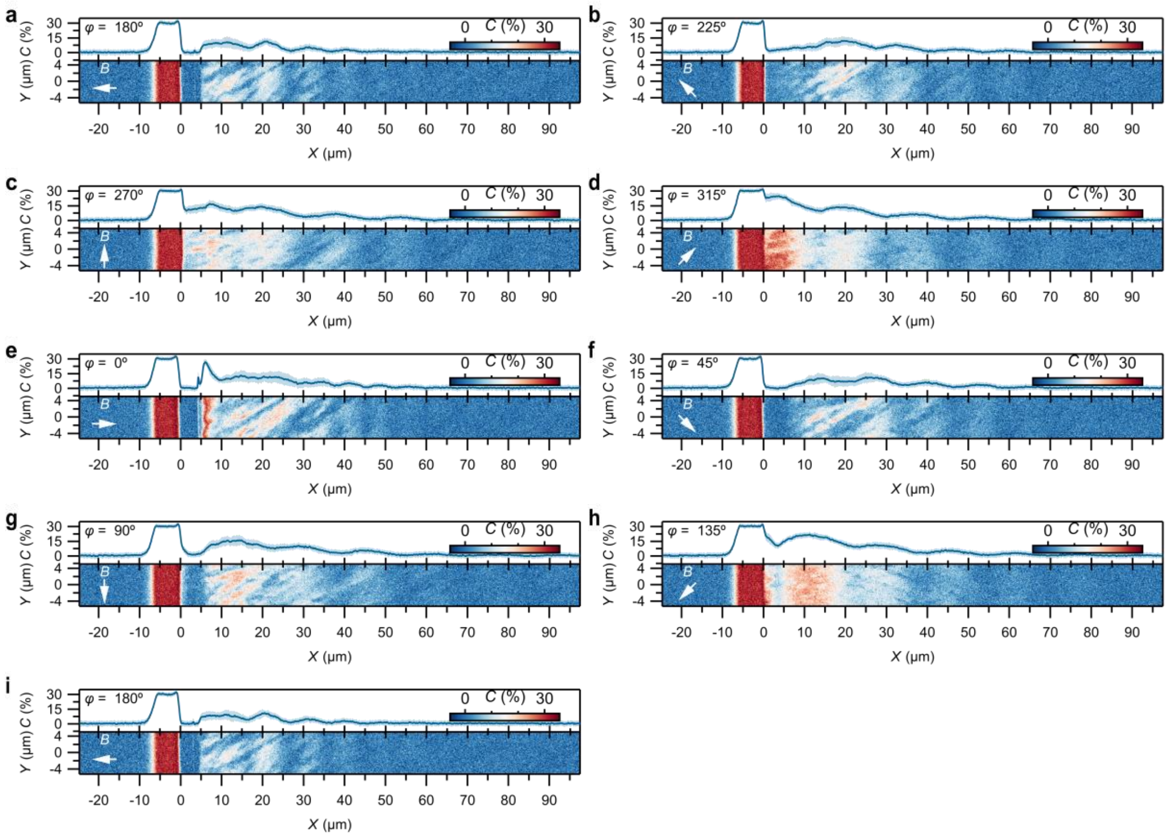

Supplementary Fig. 19. Spin-wave contrast spatial maps (bottom panel) and average profile along Y (top panel) for different in-plane applied magnetic field directions (φ). Silicon substrate, microstrip and Py film correspond to X < -5 µm, -5 < X < 0 µm, and X > 0 µm, respectively. The shaded area in the top panels corresponds to the standard deviation. The orientation of the applied magnetic field is denoted with a white arrow. The spin-waves are measured in a bimodal scheme with f = 2.869 GHz and an external applied magnetic field of 0.39 mT.

2.2.3. Microwave power dependence

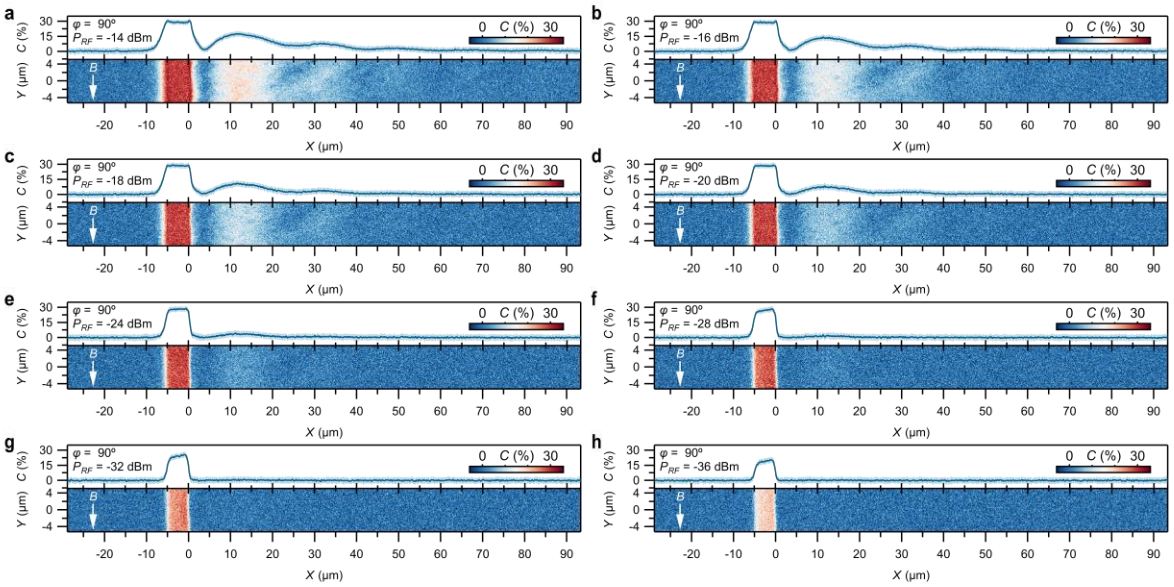

Supplementary Fig. 20. Spin-wave contrast spatial maps (bottom panel) and average profile along Y (top panel) for different applied microwave powers (denoted in every panel) and an in-plane applied magnetic field directions parallel to the microstrip (φ=90°). Silicon substrate, microstrip and Py film correspond to X < -5 µm, -5 < X < 0 µm, and X > 0 µm, respectively. The shaded area in the top panels corresponds to the standard deviation. The orientation of the applied magnetic field is denoted with a white arrow. The spin-waves are measured in a bimodal scheme with f = 2.869 GHz and an external applied magnetic field of 0.98 mT.

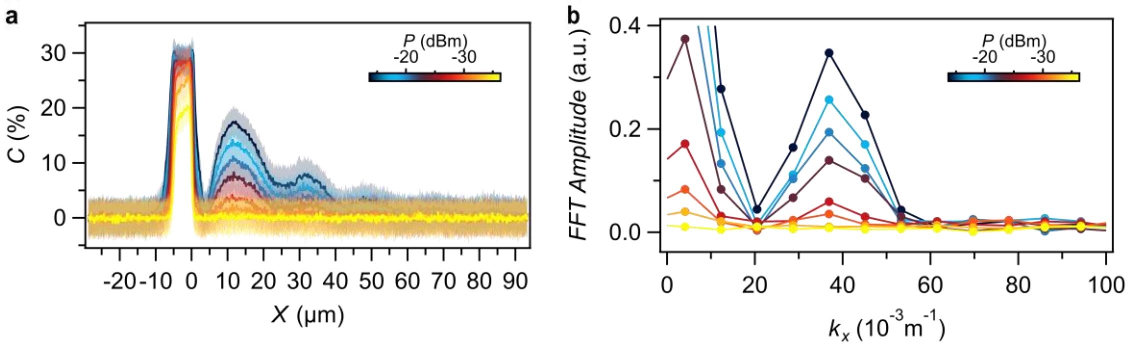

Supplementary Fig. 21. Comparison of the average spin-wave contrast profile shown in the Supplementary Figure 5 (a) and its corresponding FFT (b). The spin-waves are not observable for powers below -24 dBm.

2.2.4. Laser power dependence

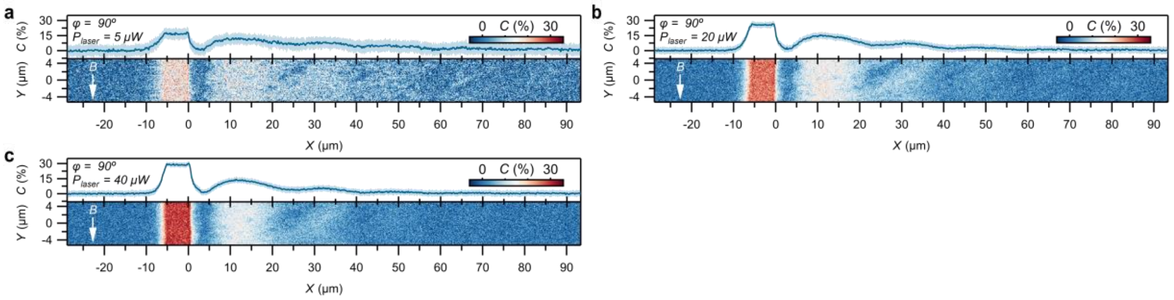

Supplementary Fig. 22. Spin-wave contrast spatial maps (bottom panel) and average profile along Y (top panel) for different optical powers (denoted in every panel) and an in-plane applied magnetic field directions parallel to the microstrip (φ=90°). Silicon substrate, microstrip and Py film correspond to X < -5 µm, -5 < X < 0 µm, and X > 0 µm, respectively. The shaded area in the top panels corresponds to the standard deviation. The orientation of the applied magnetic field is denoted with a white arrow. The spin-waves are measured in a bimodal scheme with f = 2.869 GHz and an external applied magnetic field of 0.98 mT.

2.2.5. NV-sample distance dependence

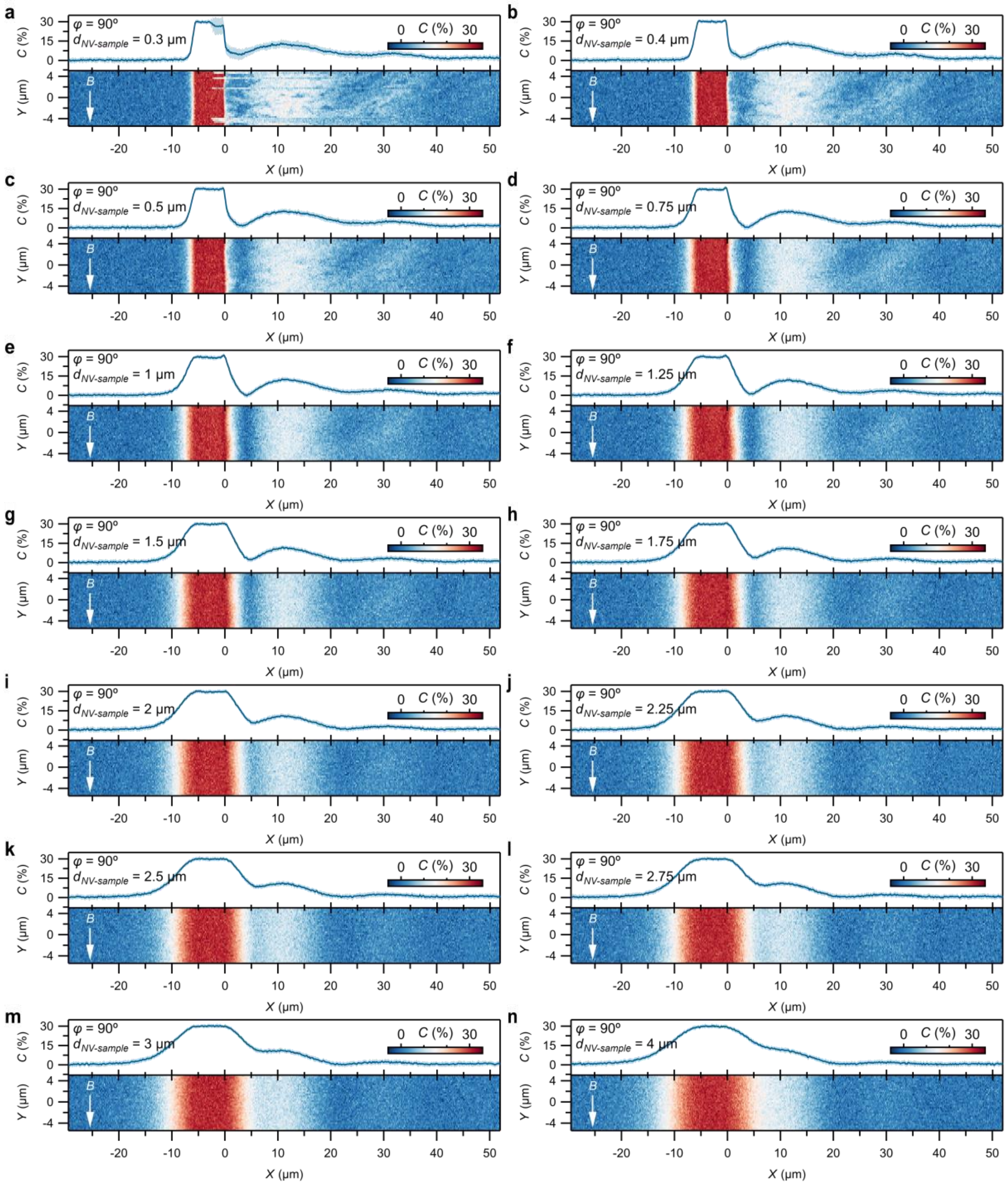

Supplementary Fig. 23. Spin-wave contrast spatial maps (bottom panel) and average profile along Y (top panel) for different tip-sample distances (denoted in every panel) and an in-plane applied magnetic field directions parallel to the microstrip (φ=90°). Silicon substrate, microstrip and Py film correspond to $X < -5$ µm, $-5 < X < 0$ µm, and $X > 0$ µm, respectively. The shaded area in the top panels corresponds to the standard deviation. The orientation of the applied magnetic field is denoted with a white arrow. The spin-waves are measured in a bimodal scheme with f = 2.869 GHz and an external applied magnetic field of 0.98 mT.

2.2.6. Field strength dependence for *k* || *B*

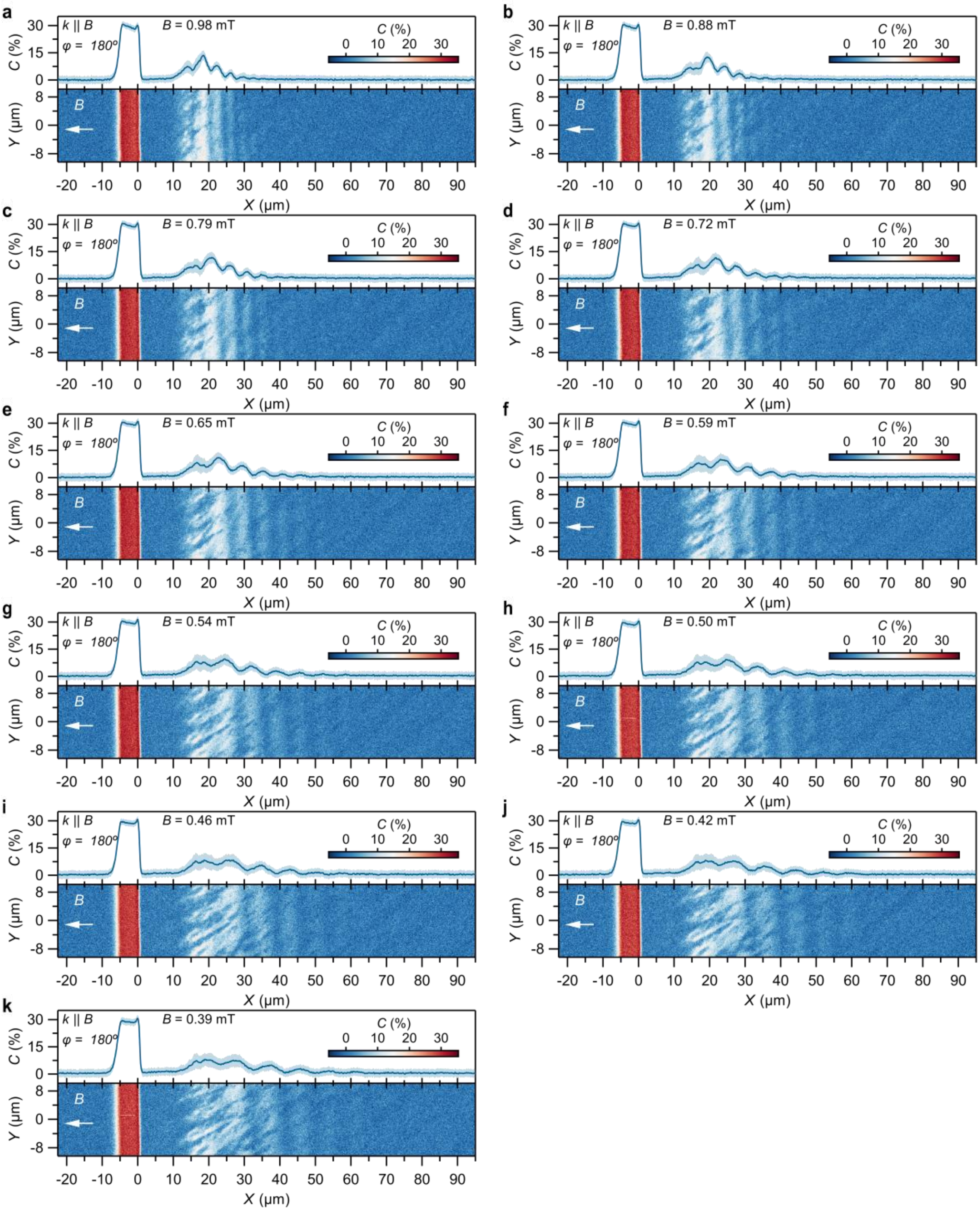

Supplementary Fig. 24. Spin-wave contrast spatial maps (bottom panel) and average profile along Y (top panel) for different external in-plane magnetic fields (denoted in every panel) applied magnetic field directions perpendicular to the microstrip (φ=180°). Silicon substrate, microstrip and Py film correspond to X < -5 µm, -5 < X < 0 µm, and X > 0 µm, respectively. The shaded area in the top panels corresponds to the standard deviation. The orientation of the applied magnetic field is denoted with a white arrow. The spin-waves are measured in a bimodal scheme with *f* = 2.869 GHz.

2.2.7. Field strength dependence for *k* ⊥ *B*

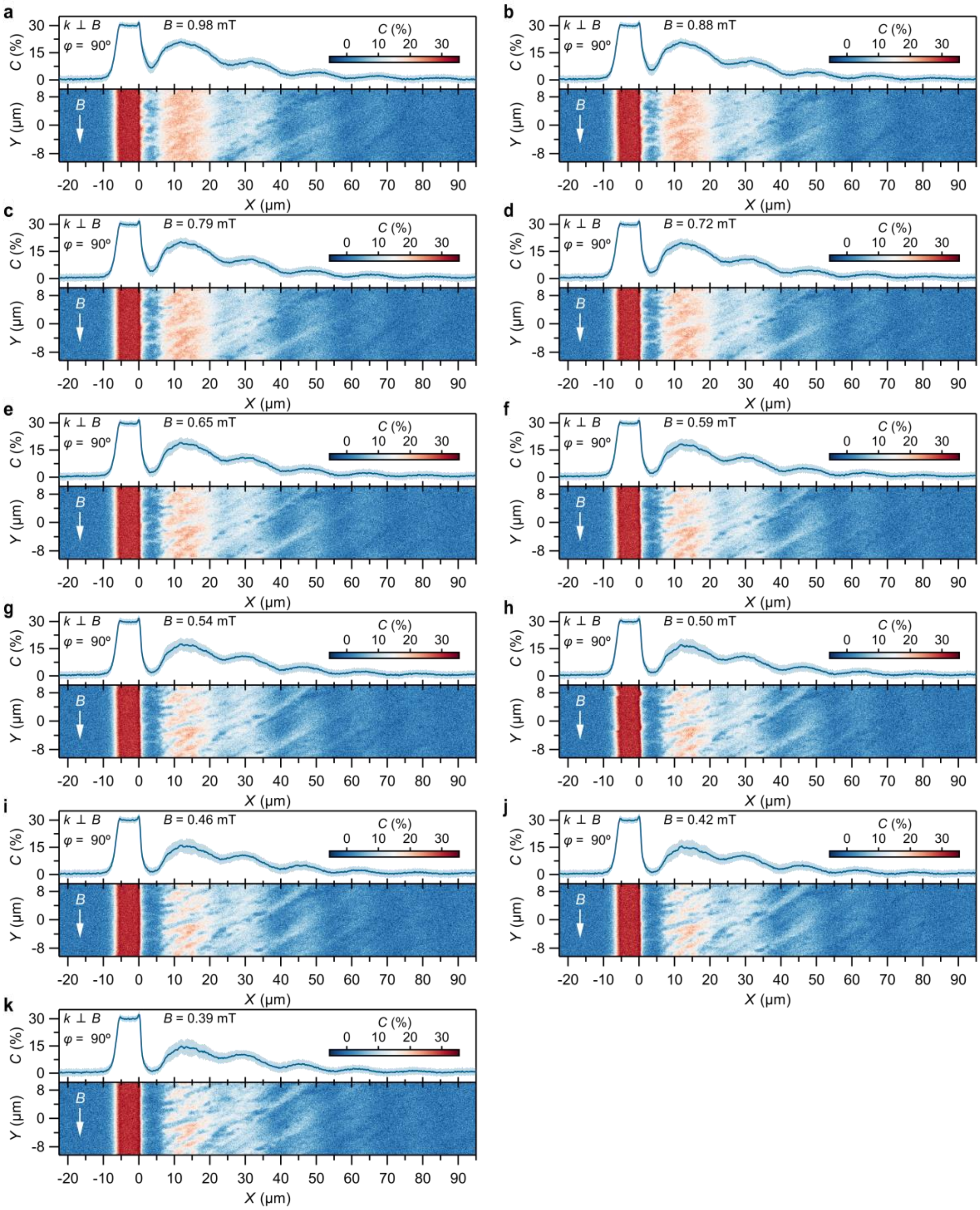

Supplementary Fig. 25. Spin-wave contrast spatial maps (bottom panel) and average profile along Y (top panel) for different external in-plane magnetic fields (denoted in every panel) applied magnetic field directions parallel to the microstrip (φ=90°). Silicon substrate, microstrip and Py film correspond to $X < -5$ µm, $-5 < X < 0$ µm, and $X > 0$ µm, respectively. The shaded area in the top panels corresponds to the standard deviation. The orientation of the applied magnetic field is denoted with a white arrow. The spin-waves are measured in a bimodal scheme with $f = 2.869$ GHz.

2.2.8. Spin wavelength evolution in a curling configuration

We observe a spatial decay of the spin wavelength in the curling state, especially for fields applied parallel to the spin wave propagation. In this section, we show its dependence for two field orientations with a magnitude of 0.98 mT: φ = 0° (**Supplementary Figure 26**) and φ = 180° (**Supplementary Figure 27**) by fitting the individual waves to a gaussian profile. The location of the centers (x_i, where i denotes the different peaks) are shown in a, a detail on the fits in b and the evolution of the wavelength (Δ_{ij}, where i and j are two consecutive peaks) in c.

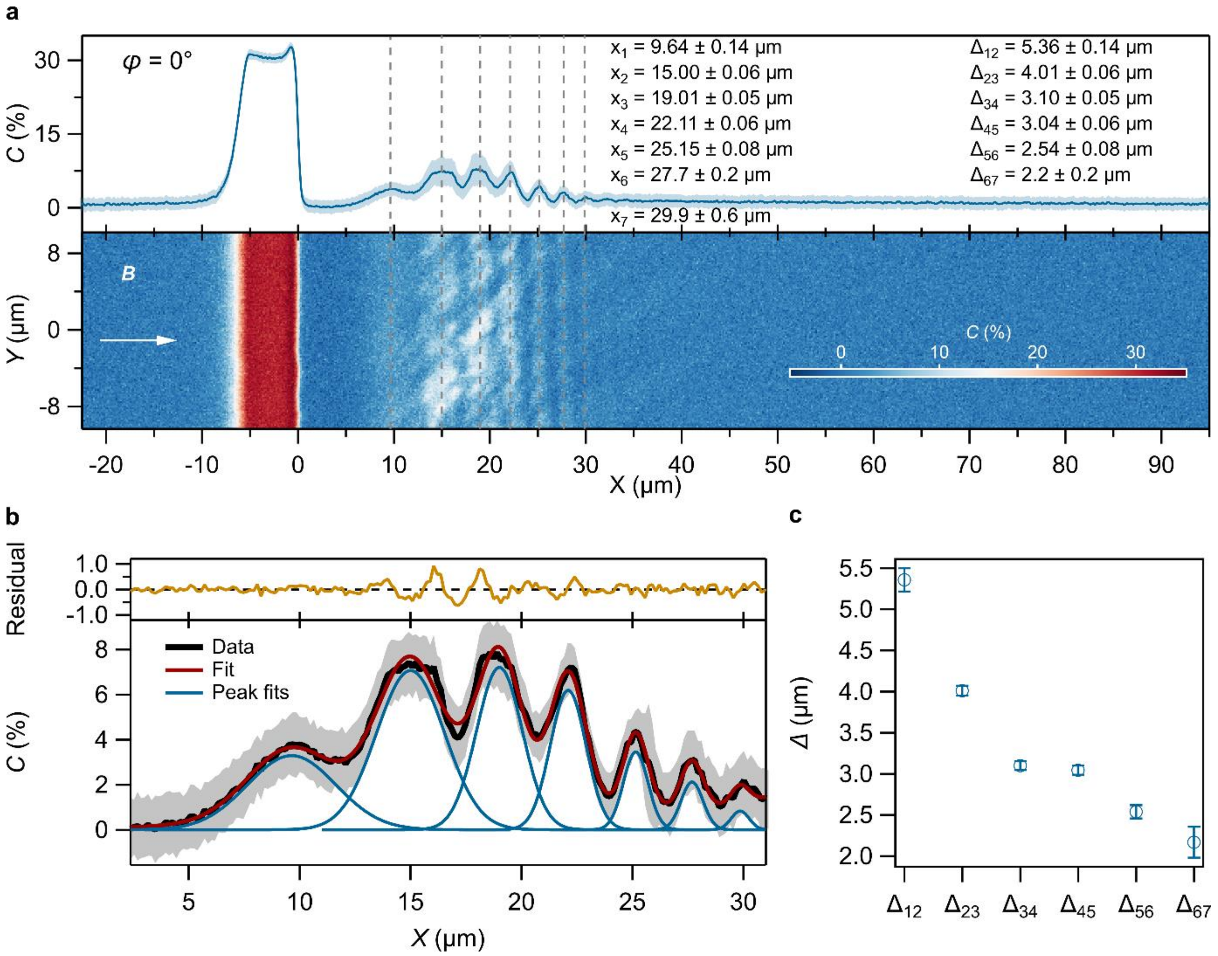

Supplementary Fig. 26. Extracting the spin wavelength evolution in a curling configuration. a) Spatial spin-wave maps (bottom panel) and their average along Y (top panel) for an in-plane field of B = 0.98 mT applied perpendicular to the microstrip (φ = 0°). Microwave drive frequency: 2.869 GHz. The bare silicon (Si) substrate, Au microstrip, and Py film are located at X < -5 μm, -5 < X < 0 μm, and X > 0 μm, respectively. Shaded area in top panel: ±1 standard deviation. White arrow: direction of the applied magnetic field. The spin wave maxima are indicated by a dashed line. b) Detail of the multi-peak fitting based on gaussian profiles and a linear background (bottom panel) together with the residual (top panel). c) Evolution of the spin wavelength considering the difference between the centers of two consecutive peaks.

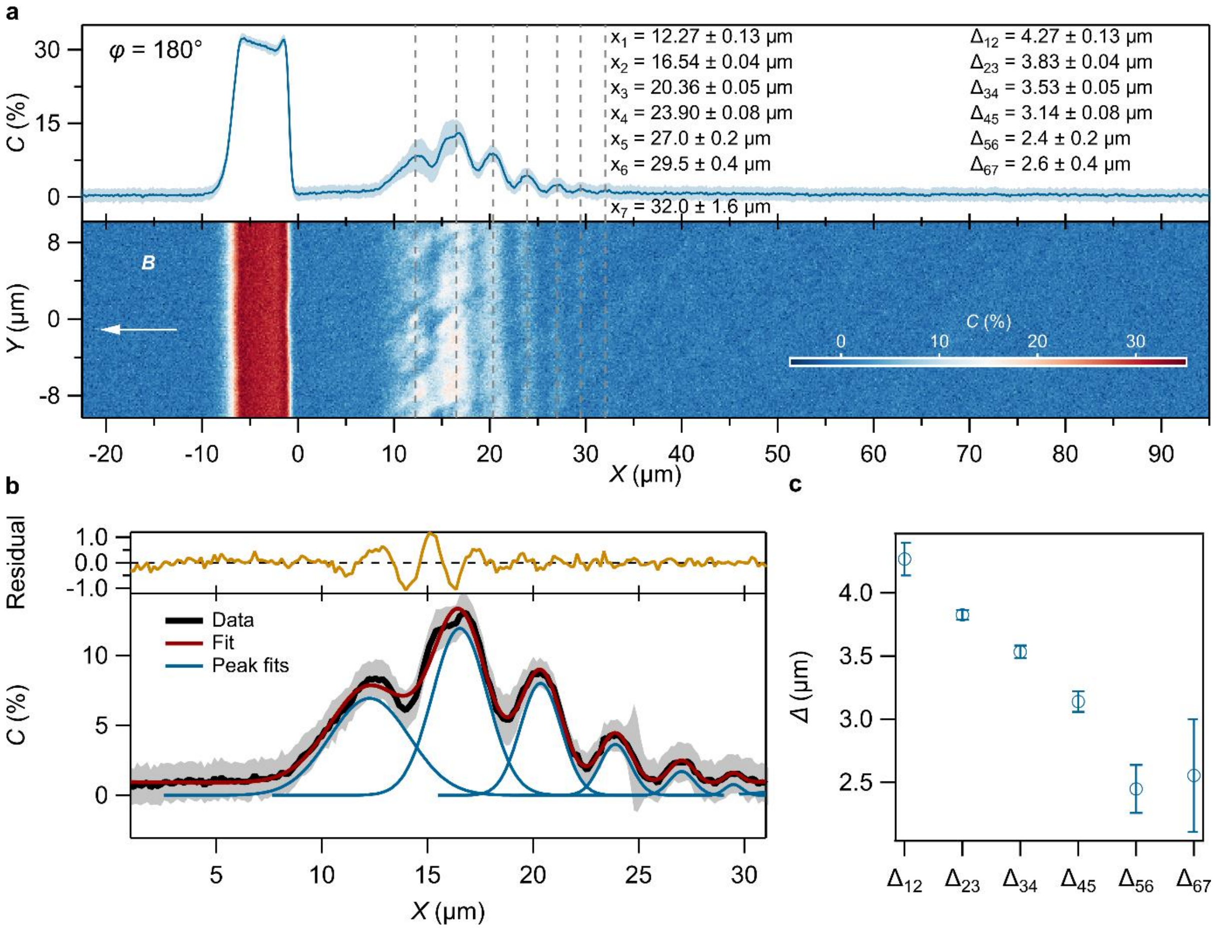

Supplementary Fig. 27. Extracting the spin wavelength evolution in a curling configuration. a) Spatial spin-wave maps (bottom panel) and their average along Y (top panel) for an in-plane field of B = 0.98 mT applied perpendicular to the microstrip (φ = 180°). Microwave drive frequency: 2.869 GHz. The bare silicon (Si) substrate, Au microstrip, and Py film are located at X < -5 µm, -5 < X < 0 µm, and X > 0 µm, respectively. Shaded area in top panel: ±1 standard deviation. White arrow: direction of the applied magnetic field. The spin wave maxima are indicated by a dashed line. b) Detail of the multi-peak fitting based on gaussian profiles and a linear background (bottom panel) together with the residual (top panel). c) Evolution of the spin wavelength considering the difference between the centers of two consecutive peaks.

2.2.9. Comparative between the theoretical and experimental spin wavelengths

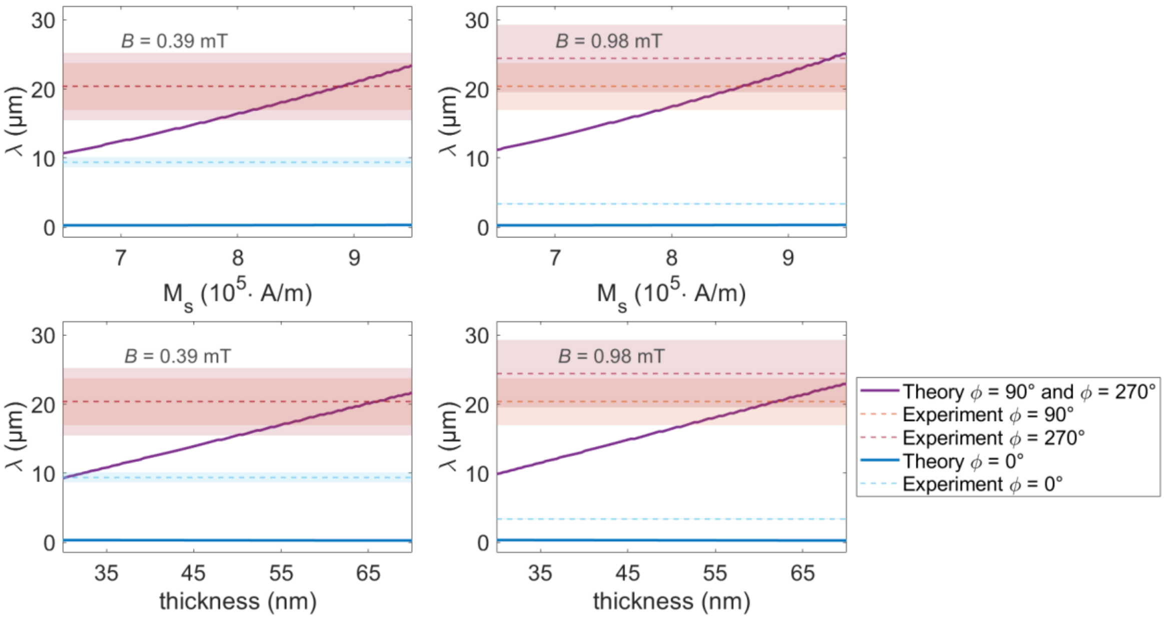

Supplementary Fig. 28. Theoretical spin wavelength as a function of the saturation magnetization, M_s, and the film thickness for different in-plane bias field angles, φ. The theoretical curves are based on the LLG equation without in-plane anisotropy (**Methods**). Dashed lines: experimentally found wavelengths, with the shaded areas indicating the standard deviation (also shown in **Fig. 3**). The theoretical spin wavelengths for φ = 90° and φ = 270° only match experiment for values of M_s that are above typical values for Py ($8\cdot 10^5$A/m),[5] or for films with a higher thickness than the measured film (53 nm). The spin wavelength for φ = 0° does not match the anisotropy-free theory for any value of Ms or thickness.

3. Electron Spin Resonance (ESR)

3.1. ESR spatial dependence for field applied parallel and perpendicular to the microstrip

In analogy with the experiments shown in **Figure 5**, we take advantage of the exquisite capabilities for sensing static magnetic fields of the NV center and perform a series of ESR spectra along a line in the X direction ranging from the silicon substrate to the permalloy film for an field of 0.98 mT applied parallel and perpendicular to the microstrip (**Supplementary Figure 29**). In agreement with the bimodal measurements (**Figure 3** and **Figure 4**), there is no ESR signal in the silicon substrate and a single broad deep centered at 2.869 GHz on top of the microstrip. Considering the permalloy area, the application of a field parallel to the microstrip (φ = 90° and φ = 270° in **Supplementary Figure 29**) is characterized by a single deep at 2.869 GHz with no significant frequency variation over the permalloy film. In contrast, a clear split is observed in the perpendicular configuration (φ = 0° and φ = 180°) next to the edge. Such symmetric splitting in the ESR is larger at the edge and shrinks over tents of micrometers until a single deep is reached. Interestingly, the ESR splitting can be quantified and yields to the field along the z-axis (top panel in **Supplementary Figure 29.a**), observing fields up to 3 mT next to edge. Upon different in-plane magnetizations, the ESR splitting next to edge is maximum in the perpendicular case (φ = 0° and φ = 180°) and progressively diminishes while rotating towards to φ = 90°/270°, as shown in the **Supplementary Section 3.3** for different in-plane angles. The observed field profile shown in **Supplementary Figure**

29.a do not exhibit a strong variation over the Y direction, as evidenced in **Supplementary Figure 29.b**.

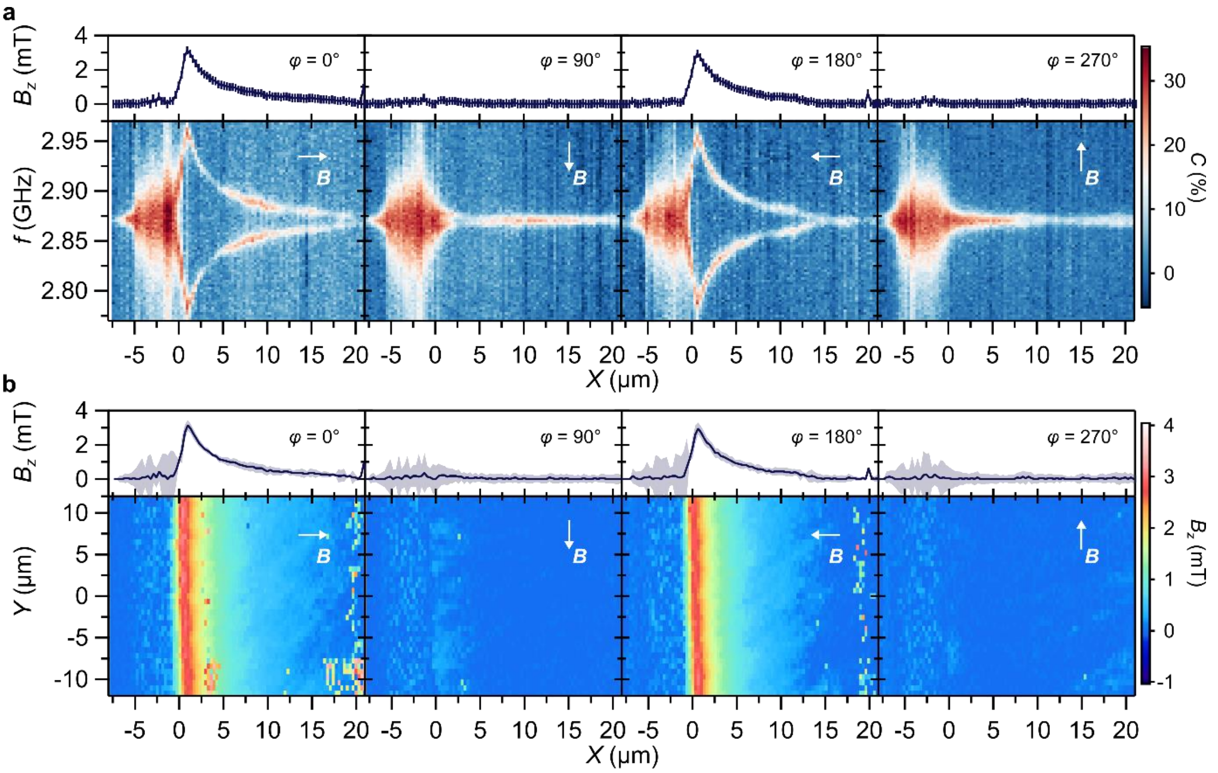

Supplementary Fig. 29. ESR imaging along the Py edge for fields perpendicular (φ = 0°/180°) and parallel (φ = 90°/270°) to the microstrip. a) ESR measurements along X (bottom panel) with the corresponding magnetic field along the z-axis (top panel) for selected in-plane applied magnetic field directions (the full angular dependence is shown in the Supplementary Section 3.2). The magnetic field strength is determined following Bz = (f+ - f-)/2γ, where gamma is the gyromagnetic ratio. b) Spatial field dependence (bottom panel) obtained from ESR profiles, as shown in a, together with average profile along Y (top panel) for different in-plane applied magnetic field directions. The field magnitude is 0.98 mT.

3.2. ESR spatial dependence for field applied perpendicular to the microstrip

As an example of the ESR imaging underlying the data shown in **Figure 5**, we show in the **Supplementary Figure 30** the ESR obtained along $Y = 0$ in a curling state (left panel) and under the formation of a domain wall (right panel). Moving from left to right across the microstrip, the small ESR contrast above the bare silicon substrate changes into a strong broad dip on the microstrip, reflecting the sensitivity of our out-of-plane NV sensor to the in-plane component of the microstrip field. Moving into the permalloy area in the counterclockwise case (left panel, **Supplementary Fig. 5b**), we observe a splitting of the NV ESR frequencies that progressively closes while, for the clockwise rotation (right panel, **Supplementary Fig. 5b**), there are several local maxima and minima. From the splitting, we extract the out-of-plane component of the magnetic field (1D profiles in **Supplementary Fig. 5b**), observing fields up to 3 mT at the film edge. These ESR maps do not vary significantly over the y direction, indicating a translationally invariant system along y (**Fig. 5c**).

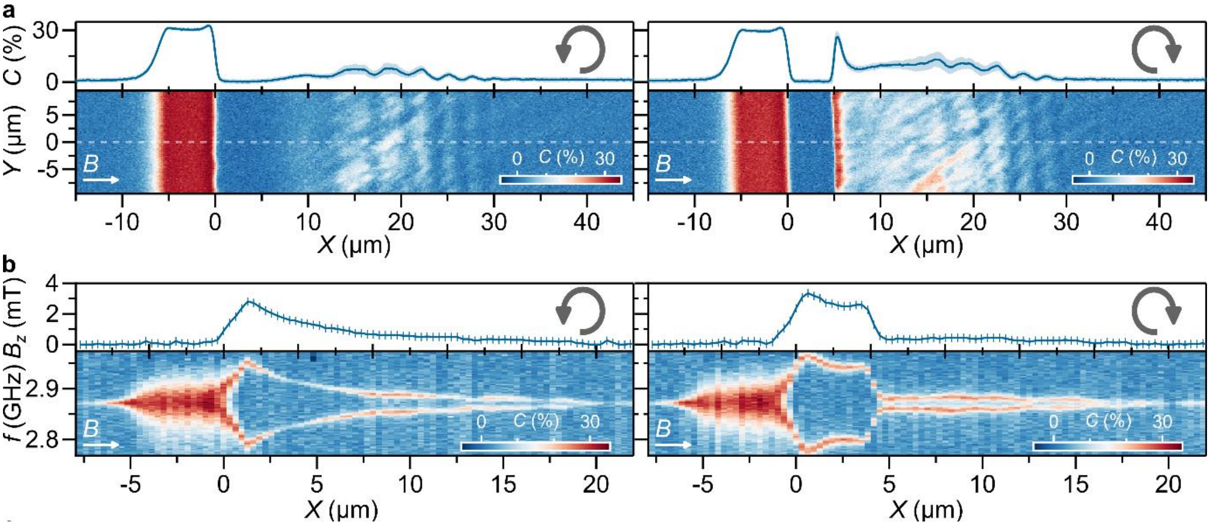

Supplementary Fig. 30. Spin-wave imaging in bistable, inhomogeneous spin textures created by magnetic-history control. **a**) Spin wave maps and their Y-averages at $B = 0.98$ mT and $f_{NV} = 2.87$ GHz with the bias field along X (white arrows). The left (right) panels are obtained after rotating the bias field counterclockwise (clockwise), as indicated by the grey arrows. **b**) Spatial NV ESR spectra measured across the Py edge (color maps) from which we extract the out-of-plane field (line traces) using $B_z = (f_+ - f_-)/2\gamma$, where γ is the gyromagnetic ratio. Measurements taken at $Y = 0$ μm (dashed line in (a)). The counterclockwise (clockwise) case shows a single (double) peak in B_z.

3.3. ESR angular dependence

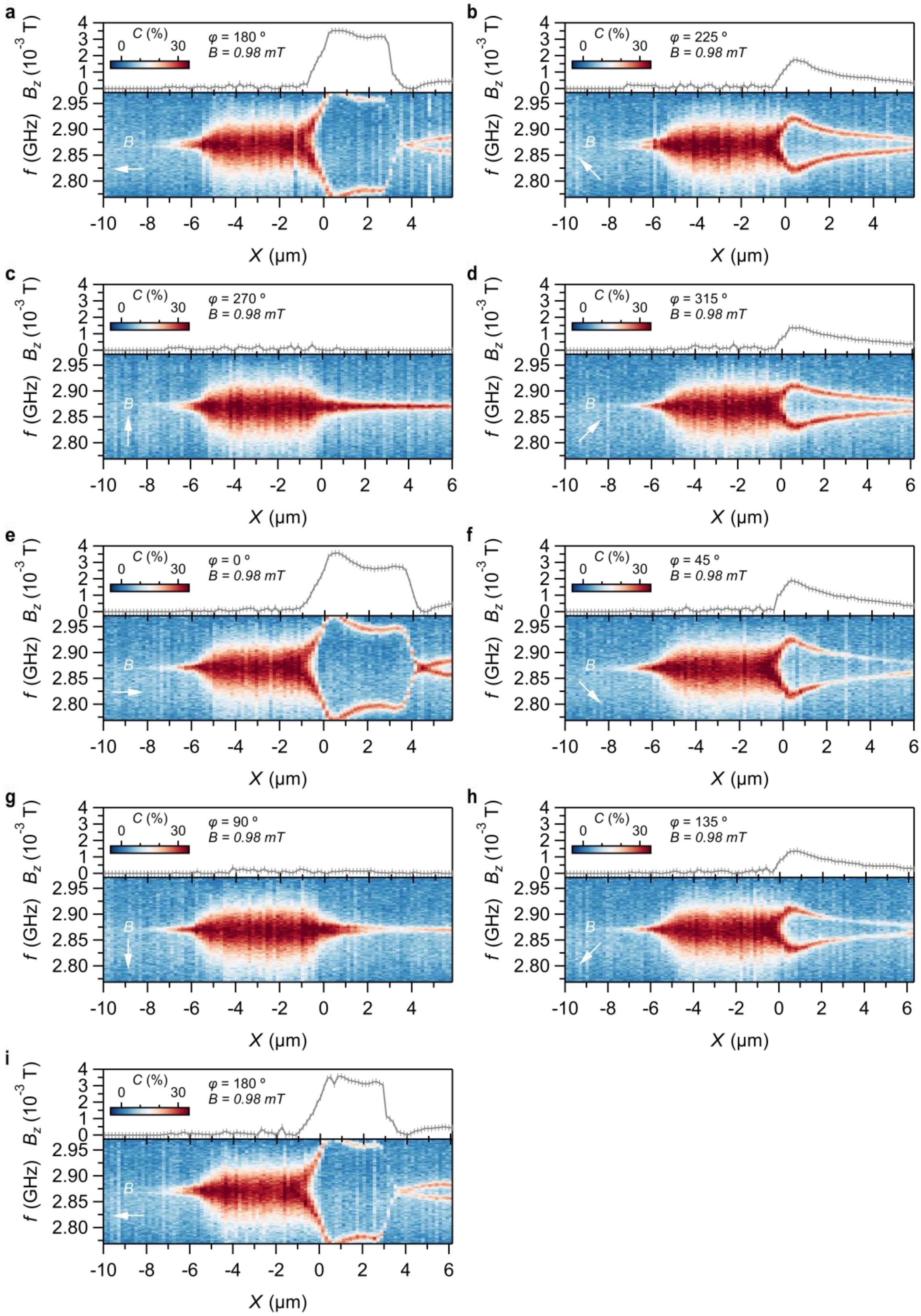

Supplementary Fig. 31. Spatial NV ESR spectra measured across the Py edge (color maps) from which we extract the out-of-plane field (line traces) using $B_z = (f_+ - f_-)/2\gamma$, where γ is the gyromagnetic ratio, for different in-plane magnetic fields (indicated by φ in the graphs) with a magnitude of 0.98 mT.

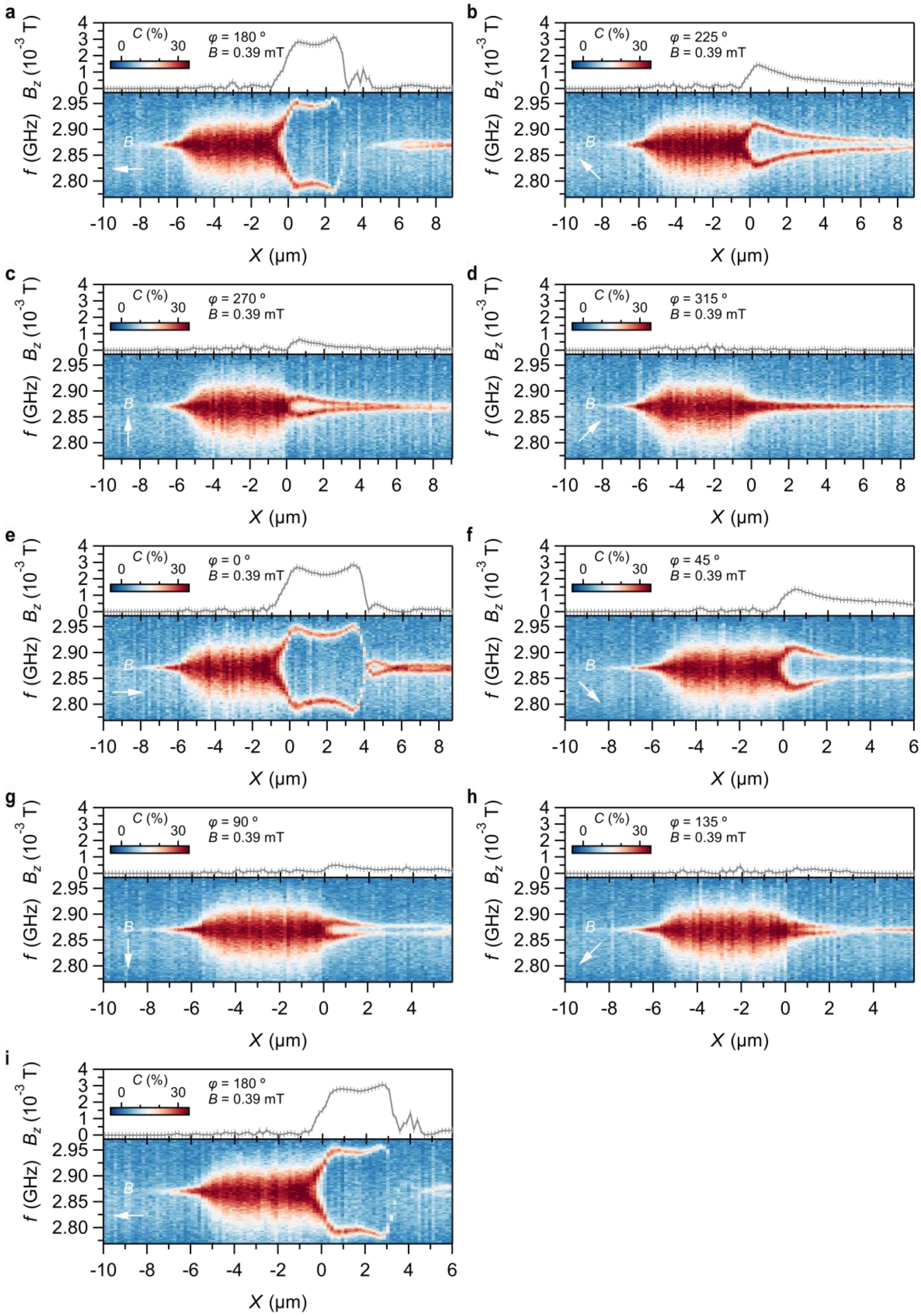

Supplementary Fig. 32. Spatial NV ESR spectra measured across the Py edge (color maps) from which we extract the out-of-plane field (line traces) using $B_z = (f_+ - f_-)/2\gamma$, where γ is the gyromagnetic ratio, for different in-plane magnetic fields (indicated by φ in the graphs) with a magnitude of 0.39 mT.

4. References

1. Ortner, M. & Coliado Bandeira, L. G. Magpylib: A free Python package for magnetic field computation. *SoftwareX* 11, 100466 (2020).
2. Hirono, S., Nonaka, K. & Hatakeyama, I. Magnetization distribution analysis in the film edge region under a homogeneous field. *J Appl Phys* 60, 3661–3670 (1986).
3. Dovzhenko, Y. *et al.* Magnetostatic twists in room-temperature skyrmions explored by nitrogen-vacancy center spin texture reconstruction. *Nat Commun* 9, 2712 (2018).
4. Landau, L.D. & Lifshitz, E. M. The electromagnetic field equiation in *The Classical Theory of Fields* (eds. Landau, L. D. & Lifshitz, E. M.) vol. 2 66–88 (Elsevier, Amsterdam, 1975).
5. Renuka Balakrishna, A. & James, R. D. A solution to the permalloy problem—A micromagnetic analysis with magnetostriction. *Appl Phys Lett* 118, 212404 (2021).
